# Mapping parameter spaces of biological switches

**DOI:** 10.1371/journal.pcbi.1008711

**Published:** 2021-02-08

**Authors:** Rocky Diegmiller, Lun Zhang, Marcio Gameiro, Justinn Barr, Jasmin Imran Alsous, Paul Schedl, Stanislav Y. Shvartsman, Konstantin Mischaikow

**Affiliations:** 1 Department of Chemical and Biological Engineering, Princeton University, Princeton, New Jersey, United States of America; 2 Lewis-Sigler Institute for Integrative Genomics, Princeton University, Princeton, New Jersey, United States of America; 3 Department of Mathematics, Rutgers, The State University of New Jersey, Piscataway, New Jersey, United States of America; 4 Instituto de Ciências Matemáticas e de Computação, Universidade de São Paulo, São Carlos, São Paulo, Brazil; 5 Department of Molecular Biology, Princeton University, Princeton, New Jersey, United States of America; 6 Flatiron Institute, Simons Foundation, New York, New York, United States of America; University of Southern California, UNITED STATES

## Abstract

Since the seminal 1961 paper of Monod and Jacob, mathematical models of biomolecular circuits have guided our understanding of cell regulation. Model-based exploration of the functional capabilities of any given circuit requires systematic mapping of multidimensional spaces of model parameters. Despite significant advances in computational dynamical systems approaches, this analysis remains a nontrivial task. Here, we use a nonlinear system of ordinary differential equations to model oocyte selection in *Drosophila*, a robust symmetry-breaking event that relies on autoregulatory localization of oocyte-specification factors. By applying an algorithmic approach that implements symbolic computation and topological methods, we enumerate all phase portraits of stable steady states in the limit when nonlinear regulatory interactions become discrete switches. Leveraging this initial exact partitioning and further using numerical exploration, we locate parameter regions that are dense in purely asymmetric steady states when the nonlinearities are not infinitely sharp, enabling systematic identification of parameter regions that correspond to robust oocyte selection. This framework can be generalized to map the full parameter spaces in a broad class of models involving biological switches.

## Author summary

Identification of qualitatively different regimes in models of biomolecular switches is essential for understanding dynamics of complex biological processes, including symmetry breaking in cells and cell networks. We demonstrate how topological methods, symbolic computation, and numerical simulations can be combined for systematic mapping of symmetry-broken states in a mathematical model of oocyte specification in *Drosophila*, a leading experimental system of animal oogenesis. Our algorithmic framework reveals global connectedness of parameter domains corresponding to robust oocyte specification and enables systematic navigation through multidimensional parameter spaces in a large class of biomolecular switches.

## Introduction

Clusters of cells interconnected by stable cytoplasmic bridges serve important functions in existing species and are thought to have played a role in the emergence of multicellularity [1]. A notable example wherein such cysts arise is animal oogenesis, which includes an obligate stage where the future oocyte develops while connected to auxiliary cells that supply it with molecules and organelles [2–8]. Here we focus on dynamics within the female germline cyst in *Drosophila*, one of the leading experimental systems for studies of oogenesis [9].

**Fig 1:**
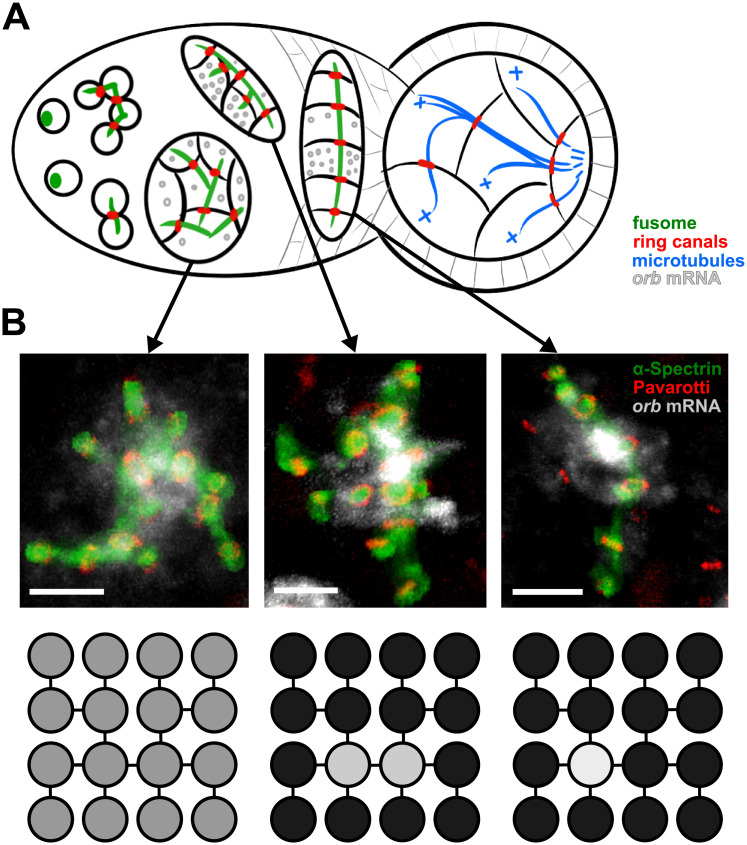
Dynamics of oocyte determination. (A) During the cyst’s progression through the germarium, the oocyte-specifying factor Orb is initially produced in all cells, but then localizes to the two central cells, and finally to a single cell, the future oocyte. Throughout this process, the fusome (green) forms a backbone within the cyst, leading to the formation of polarized microtubules terminating in the oocyte. (B) Confocal images of the fusome (*α*-Spectrin, green) and ring (Pavarotti, red) backbone that lies within the network of cells, and corresponding 16-cell schematic denoting the progression of *orb* mRNA (gray), from a uniform distribution throughout all cells, to localization within the two central cells, and finally to the oocyte (scale bar = 5 *μ*m).

The developmental unit of *Drosophila* oogenesis is the egg chamber—a 16-cell germline cyst surrounded by a somatically derived epithelium [3]. This cyst arises from a single stem cell-derived cystoblast (Fig 1A) that undergoes four synchronous divisions, generating the stereotypical network of cells shown in Fig 1B [9]. These divisions are incomplete, however, leaving cells interconnected by reinforced cytoplasmic bridges called ring canals [10, 11] that allow for transport of proteins, mRNA, and cytoplasmic components between cells. The invariant pattern of divisions generates a lineage tree with bilateral symmetry. The oocyte differentiates from one of the two central cells with 4 ring canals. The 15 remaining cells become nurse cells, supplying the oocyte with components required for its growth and development [12].

Only one cell within the cyst must be selected as the future oocyte. The selection of a single cell as the oocyte corresponds to the proper dynamic localization of many factors, such as BicD, Egl, and dynein [13, 14]. Another key component of this selection process is Orb, an important mRNA-binding translational regulator of multiple transcripts in the developing egg chamber that is necessary for establishing polarity within the developing cyst [15–19]. In early stages of development, this factor is expressed uniformly in low amounts within all cells of the cyst. However, as oogenesis progresses, *orb* mRNA begins to migrate to the two central cells with four ring canals, and finally to just a single cell, the eventual oocyte (see Fig 1B) [20]. Notably, Orb promotes its own translation and indirectly affects transport of its own transcript through ring canals [21, 22].

Two models have been proposed to explain the differentiation of the oocyte from all other cells in the cyst: prepatterning (biased) and self-organizing (unbiased). The biased mechanism relies on the nonuniform distribution of the fusome, a membranous structure that is generated during cyst formation and passes through the 15 ring canals in the 16-cell cyst [13, 23]. This backbone is formed progressively with every sequential division and is critical for synchronized mitotic divisions and intercellular communication within the cyst [9]. According to this model, the future oocyte is predetermined by the asymmetric distribution of fusome material during the first cell division, well before the entire cyst is fully formed [14].

In contrast, the unbiased model postulates that either of the two central cells can be selected as the oocyte, independently of fusome asymmetry [9]. Because Orb protein binds to the untranslated region of its own mRNA (3’UTR) and positively promotes mRNA translation, a feedback loop is established that alters the intercellular transport of *orb* mRNA between the two cells in order to select the future oocyte [20–22]. Recent work has provided evidence that the fusome does not play as direct a role in oocyte specification, but instead aides Orb in localizing *orb* mRNA to one of the cells, therefore polarizing the developing cyst [20]. This would suggest that either of the central cells can become enriched for *orb* mRNA and Orb protein, eventually leading to its selection as the oocyte. Here, we evaluate this autoregulatory mechanism using a mathematical model and demonstrate that it enables robust oocyte selection without preexisting bias.

### Model for oocyte selection in *Drosophila*

**Fig 2:**
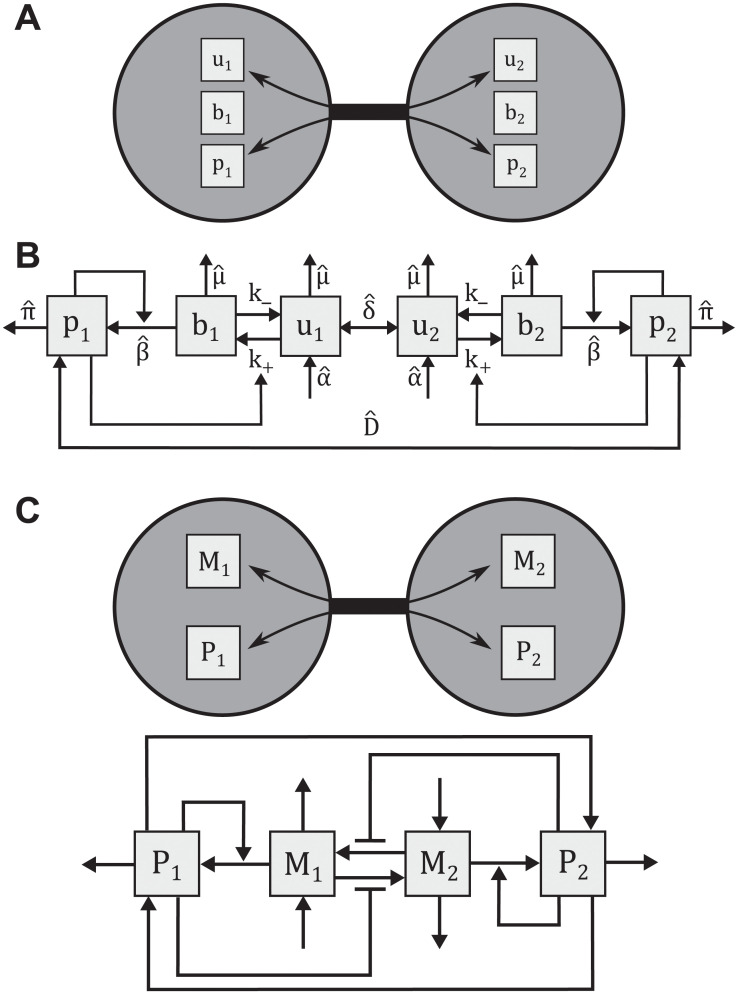
The oocyte selection model. (A) Overview of species in the model. *orb* mRNA that has been bound (*b*) to fusome does not exchange between cells, unbound *orb* mRNA (*u*) and Orb protein (*p*) are not restricted in their movement. (B) Schematic of unbound *orb* mRNA (*u*_1,2_), bound *orb* mRNA (*b*_1,2_), and Orb protein (*p*_1,2_) interactions between the two most central cells. (C) Regulatory interactions in the reduced model.

Although initially present in all 16 cells of the cyst, *orb* mRNA eventually localizes to the two central cells in the cyst (Fig 1). This allows for the 16-cell model to be reduced to just the two central cells. To model oocyte selection, we consider a localization mechanism where Orb protein in each central cell binds freely diffusing *orb* mRNA to its fusome. A summary of this process is shown in Fig 2A. In this case, the dynamics of concentrations for unbound (*u*_1_, *u*_2_) and bound (*b*_1_, *b*_2_) forms of mRNA in each cell, as well as the respective concentrations of Orb protein (*p*_1_, *p*_2_) can be represented by the following six-dimensional dynamical system:

$$\begin{array}{c} \frac{du_{1}}{d\tau }=\hat{\alpha }-\hat{\mu }u_{1}+k_{-}b_{1}-(k_{+,0}+k_{+,\Delta }\frac{p_{1}^{n}}{p_{1}^{n}+\Theta_{1}^{n}})u_{1}+\hat{\delta }\left(u_{2}-u_{1}\right), \end{array}$$ (1)

$$\begin{array}{c} \frac{du_{2}}{d\tau }=\hat{\alpha }-\hat{\mu }u_{2}+k_{-}b_{2}-(k_{+,0}+k_{+,\Delta }\frac{p_{2}^{n}}{p_{2}^{n}+\Theta_{1}^{n}})u_{2}+\hat{\delta }\left(u_{1}-u_{2}\right), \end{array}$$ (2)

$$\begin{array}{c} \frac{db_{1}}{d\tau }=(k_{+,0}+k_{+,\Delta }\frac{p_{1}^{n}}{p_{1}^{n}+\Theta_{1}^{n}})u_{1}-k_{-}b_{1}-\hat{\mu }b_{1}, \end{array}$$ (3)

$$\begin{array}{c} \frac{db_{2}}{d\tau }=(k_{+,0}+k_{+,\Delta }\frac{p_{2}^{n}}{p_{2}^{n}+\Theta_{1}^{n}})u_{2}-k_{-}b_{2}-\hat{\mu }b_{2}. \end{array}$$ (4)

$$\begin{array}{c} \frac{dp_{1}}{d\tau }=(\hat{\beta }+\hat{\gamma }\frac{p_{1}^{\nu }}{p_{1}^{\nu }+\Theta_{2}^{\nu }})b_{1}-\hat{\pi }p_{1}+\hat{D}\left(p_{2}-p_{1}\right), \end{array}$$ (5)

$$\begin{array}{c} \frac{dp_{2}}{d\tau }=(\hat{\beta }+\hat{\gamma }\frac{p_{2}^{\nu }}{p_{2}^{\nu }+\Theta_{2}^{\nu }})b_{2}-\hat{\pi }p_{2}+\hat{D}\left(p_{1}-p_{2}\right). \end{array}$$ (6)

The definitions of each parameter in this system are given in Table 1, and a schematic of species interactions is shown in Fig 2B.

**Table 1:** Model parameters.

Parameter	Definition
$\hat{\alpha }$	rate of mRNA transcription
$\hat{\mu }$	rate constant for mRNA degradation
*k*_−_	rate constant for mRNA unbinding
*k*_+,0_	basal rate constant of mRNA binding
*k*_+,Δ_	protein-induced increase of binding rate constant
$\hat{\delta }$	rate constant for intercellular mRNA exchange
$\hat{\beta }$	basal rate of protein translation
$\hat{\gamma }$	protein-induced increase of translation rate constant
$\hat{\pi }$	rate constant for protein degradation
$\hat{D}$	rate constant for intercellular protein exchange
Θ_1_	threshold for protein-induced mRNA binding
Θ_2_	threshold for protein-induced translation
*n*	Hill exponent for protein-induced mRNA binding
*ν*	Hill exponent for protein-induced translation

### Nondimensionalization

Let *m*_*i*_ denote the sum of bound and unbound mRNA species in a given cell *i*, where *i* = 1, 2. When binding and unbinding processes are fast compared to the time scale of oocyte selection, a rapid equilibrium approximation can be used to express the bound and unbound concentrations in terms of *m* as $m_{i}=u_{i}(1+K_{0}+K\frac{p_{i}^{n}}{p_{i}^{n}+\Theta_{1}^{n}})$, where $K_{0}=\frac{k_{+,0}}{k_{-}}\;\text{and}\;K=\frac{k_{+,\Delta }}{k_{-}}$. This can be used to reduce the number of variables from six to four:

$$\begin{array}{c} \frac{dm_{1}}{d\tau }=\hat{\alpha }-\hat{\mu }m_{1}-\hat{\delta }(\frac{1}{1+K_{0}+K\frac{p_{1}^{n}}{p_{1}^{n}+\Theta_{1}^{n}}})m_{1}+\hat{\delta }(\frac{1}{1+K_{0}+K\frac{p_{2}^{n}}{p_{2}^{n}+\Theta_{1}^{n}}})m_{2}, \end{array}$$ (7)

$$\begin{array}{c} \frac{dm_{2}}{d\tau }=\hat{\alpha }-\hat{\mu }m_{2}+\hat{\delta }(\frac{1}{1+K_{0}+K\frac{p_{1}^{n}}{p_{1}^{n}+\Theta_{1}^{n}}})m_{1}-\hat{\delta }(\frac{1}{1+K_{0}+K\frac{p_{2}^{n}}{p_{2}^{n}+\Theta_{1}^{n}}})m_{2}, \end{array}$$ (8)

$$\begin{array}{c} \frac{dp_{1}}{d\tau }=(\hat{\beta }+\hat{\gamma }\frac{p_{1}^{\nu }}{p_{1}^{\nu }+\Theta_{2}^{\nu }})(\frac{K_{0}+K\frac{p_{1}^{n}}{p_{1}^{n}+\Theta_{1}^{n}}}{1+K_{0}+K\frac{p_{1}^{n}}{p_{1}^{n}+\Theta_{1}^{n}}})m_{1}-\hat{\pi }p_{1}+\hat{D}\left(p_{2}-p_{1}\right), \end{array}$$ (9)

$$\begin{array}{c} \frac{dp_{2}}{d\tau }=(\hat{\beta }+\hat{\gamma }\frac{p_{2}^{\nu }}{p_{2}^{\nu }+\Theta_{2}^{\nu }})(\frac{K_{0}+K\frac{p_{2}^{n}}{p_{2}^{n}+\Theta_{1}^{n}}}{1+K_{0}+K\frac{p_{2}^{n}}{p_{2}^{n}+\Theta_{1}^{n}}})m_{2}-\hat{\pi }p_{2}+\hat{D}\left(p_{1}-p_{2}\right). \end{array}$$ (10)

To nondimensionalize the model, we rescale (7)–(10) time by the maximum rate of mRNA transport between cells, $t=\hat{\delta }\tau /\left(1+K_{0}\right)$, mRNA by the ratio of the rate of mRNA formation to the maximum rate of intercellular mRNA transport, $M_{i}=\hat{\delta }m_{i}/\left(\hat{\alpha }\left(1+K_{0}\right)\right)$, and protein by the basal ratio of the translation of bound mRNA to the rate of unbinding and maximum transport of free mRNA, $P_{i}={\hat{\delta }}^{2}p_{i}/\left(\hat{\alpha }\hat{\beta }K_{0}\left(1+K_{0}\right)\right)$. The rescaled model takes the following form:

$$\begin{array}{c} \frac{dM_{1}}{dt}=1-\mu M_{1}-(\frac{1}{1+\kappa \frac{P_{1}^{n}}{P_{1}^{n}+\theta_{1}^{n}}})M_{1}+(\frac{1}{1+\kappa \frac{P_{2}^{n}}{P_{2}^{n}+\theta_{1}^{n}}})M_{2}, \end{array}$$ (11)

$$\begin{array}{c} \frac{dM_{2}}{dt}=1-\mu M_{2}+(\frac{1}{1+\kappa \frac{P_{1}^{n}}{P_{1}^{n}+\theta_{1}^{n}}})M_{1}-(\frac{1}{1+\kappa \frac{P_{2}^{n}}{P_{2}^{n}+\theta_{1}^{n}}})M_{2}, \end{array}$$ (12)

$$\begin{array}{c} \frac{dP_{1}}{dt}=(1+\gamma \frac{P_{1}^{\nu }}{P_{1}^{\nu }+\theta_{2}^{\nu }})(\frac{1+\eta \frac{P_{1}^{n}}{P_{1}^{n}+\theta_{1}^{n}}}{1+\kappa \frac{P_{1}^{n}}{P_{1}^{n}+\theta_{1}^{n}}})M_{1}-\pi P_{1}+\epsilon \left(P_{2}-P_{1}\right), \end{array}$$ (13)

$$\begin{array}{c} \frac{dP_{2}}{dt}=(1+\gamma \frac{P_{2}^{\nu }}{P_{2}^{\nu }+\theta_{2}^{\nu }})(\frac{1+\eta \frac{P_{2}^{n}}{P_{2}^{n}+\theta_{1}^{n}}}{1+\kappa \frac{P_{2}^{n}}{P_{2}^{n}+\theta_{1}^{n}}})M_{2}-\pi P_{2}+\epsilon \left(P_{1}-P_{2}\right). \end{array}$$ (14)

While *ν* and *n* are unchanged upon rescaling, eight dimensionless groups arise, which are defined in Table 2. Though the presented simplified model does not account for the full intricacies of interactions between *orb* mRNA and cytoskeleton within each cell, the regulatory effects of protein interactions preserve the qualitative behaviors of the system. Here, we demonstrate that this model can predict the emergence of alternative symmetry-broken states, where—depending on initial conditions—one cell accumulates more mRNA and protein. In fact, it can be shown through direct substitution that this system will have equal concentrations of total mRNA in each cell if and only if there are equal amounts of Orb protein within each cell. Therefore, in searching for symmetry broken solutions, it will suffice to look for steady states where the amount of protein within each cell at steady state is asymmetric.

**Table 2:** Dimensionless parameters.

Parameter	Definition	Interpretation
*μ*	$\frac{\hat{\mu }\left(1+K_{0}\right)}{\hat{\delta }}$	ratio of timescales of unbound mRNA transport to mRNA degradation
*π*	$\frac{\hat{\pi }\left(1+K_{0}\right)}{\hat{\delta }}$	ratio of timescales of unbound mRNA transport to protein degradation
*ϵ*	$\frac{\hat{D}\left(1+K_{0}\right)}{\hat{\delta }}$	ratio of timescales of unbound mRNA transport to protein transport
*γ*	$\frac{\hat{\gamma }}{\hat{\beta }}$	ratio of protein-induced rate of translation due to basal rate of protein translation
*θ*_1_	$\frac{\Theta_{1}{\hat{\delta }}^{2}}{\hat{\alpha }\hat{\beta }K_{0}\left(1+K_{0}\right)}$	threshold for inhibition of unbound mRNA transport
*θ*_2_	$\frac{\Theta_{2}{\hat{\delta }}^{2}}{\hat{\alpha }\hat{\beta }K_{0}\left(1+K_{0}\right)}$	threshold for autocatalytic protein translation
*κ*	$\frac{K}{1+K_{0}}$	ratio of protein-induced binding rate of mRNA to basal rate of mRNA binding and unbinding
*η*	$\frac{K}{K_{0}}$	ratio of protein-induced binding rate of mRNA to basal rate of mRNA binding

The feedback loops in the model introduce nonlinear terms that induce switch-like behaviors. That is, this model predicts different potential outcomes for oocyte selection based on the amounts of protein in each cell relative to some threshold. Models with sharp switches have been analyzed using various approaches, such as parameter sampling [24] or by transforming the nonlinearity to a logoid function [25, 26]. Here, we adopt a different perspective that focuses on the identification of *attracting regions*, which we define as regions of phase space that are mapped onto themselves.

By replacing the sigmoidal nonlinearities (Hill functions) in Eqs (11)–(14) by step functions, we adopt a combinatorial approach to the dynamics of the system, while maintaining a continuous parameterization. In turn, this allows us to determine *a priori* the regions of parameter space that contain the same configurations of steady state behaviors. Algorithmic implementation of this strategy, called Dynamic Signatures Generated by Regulatory Networks (DSGRN), enumerates these regions of parameter space and identifies the associated dynamics [27, 28]. In contrast to numerical continuation or parameter sampling, this approach is guaranteed to identify all qualitatively different behaviors [29] and can be applied to systems with large numbers of parameters [30, 31]. A brief summary of this approach is given by the following series of steps:

1. Identify all parameters in the dynamical system of interest.
2. Convert Hill functions to sharp switches (Heaviside functions) in order to make the system piecewise linear.
3. Decompose state space based on whether each switch in the model is “on” or “off” and solve the resulting linear system within each region of state space to identify their governing inequalities with respect to system parameters.
4. Find each realizable steady state configuration by taking the union of all parametric inequalities and identifying which are non-empty sets.
5. Divide parameter space based on these parametric inequalities and identify adjacencies between distinct regions in parameter space.

### Illustrative example

**Fig 3:**
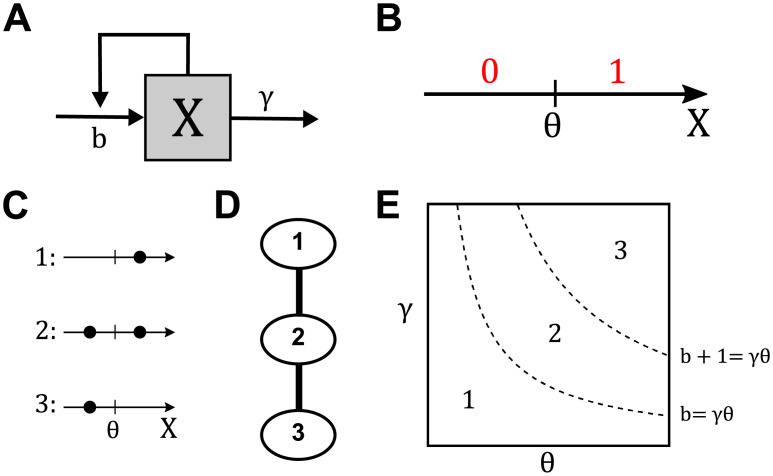
Analysis of toy model. (A) Single-variable model. (B) Division of *X* into 2 regions based on the value of *θ*. (C) All distributions of steady states in the sharp switch limit. (D) Adjacency relations of parameter regions defined in (C). (E) Visualization of parameter space projected onto (*θ*, *γ*) space for some fixed *b*. Here, we can explicitly see how parameter space has been divided based on the parametrically-derived steady state configurations.

To better illustrate the algebraic decomposition framework, we first look at a simple example. Consider a single autocatalytic component, *X*, as shown in Fig 3A. If this component has a basal rate of production, *b*, degree of self-activation, *n*, with some associated threshold, *θ*, and degradation rate, *γ*, then its dynamics can be described in the following way, for positive *X*, *b*, *θ*, *γ* and *n* > 1:

$$\begin{array}{c} \frac{dX}{dt}=(b+\frac{X^{n}}{\theta^{n}+X^{n}})-\gamma X. \end{array}$$ (15)

In the limit when *n* → ∞, where the Hill nonlinearity becomes a Heaviside function [32], the differential equation becomes:

$$\begin{array}{c} \frac{dX}{dt}=(b+H(X-\theta ))-\gamma X. \end{array}$$ (16)

This piecewise linear model can be studied using the algebraic decomposition framework, identifying attracting regions as a function of parameter values [27]. Here, (16) naturally leads to the division of phase space into 2 regions, as shown in Fig 3B: *A*_0_ = {*X*|*X* < *θ*} and *A*_1_ = {*X*|*X* > *θ*} where each distinct region is defined as a different case for the Heaviside function taking the value 0 or 1. Note that (16) in regions *A*_0_ and *A*_1_ is given, respectively, by:

$$\begin{array}{c} \frac{dX}{dt}=b-\gamma X, \end{array}$$ (17)

$$\begin{array}{c} \frac{dX}{dt}=(b+1)-\gamma X. \end{array}$$ (18)

Eqs (17) and (18) have stable steady states *f*_0_ = *b*/*γ* and *f*_1_ = (*b* + 1)/*γ*, respectively. Furthermore, if *f*_0_ ∈ *A*_0_, then *A*_0_ is an attracting region (the same is true for *A*_1_ if *f*_1_ ∈ *A*_1_). We focus on attracting regions for the following reasons: First, the actual value of the fixed point *f*_0_ is of little biological interest as it is specific to (17), which is an unrealistic limiting equation for (15). Second, the existence of a fixed point in *A*_0_ if *A*_0_ is an attracting region can be deduced from topological fixed point theorems. Finally, the property of being an attracting region is determined by the behavior of the vector field at the boundary of the region. The only requirement for a region to be attracting is that the vector field points inside along the boundary of that region. Hence if region *A*_0_ is attracting for a vector field *f*, it will also be attracting for any other vector field *g* that is not too far from *f* along the boundary of *A*_0_ (all that is required is that *g* also points inside along the boundary of *A*_0_). This suggests that the assumption that *A*_0_ can be associated with an attracting region is valid for a much broader class of equations than just (16). Importantly, this does not require the assumption that the nonlinearities of (15) are analytically precise.

Returning to Eqs (17) and (18), observe that *A*_0_ is an attracting region if and only if *b*/*γ* < *θ*, and similarly, *A*_1_ is an attracting region if and only if (*b* + 1)/*γ* > *θ*. Simple algebraic decomposition of these two inequalities allows identification of the full distribution of attracting regions or equivalently stable steady states. This decomposition is shown in Fig 3C, where all possible combinations of steady states are shown graphically. Therefore, the entire parameter space $\left(b,\theta ,\gamma \right)\in R_{+}^{3}$ can be decomposed as follows: *D*_1_ = {*b* > *γθ*}, *D*_2_ = {*b* < *γθ* < *b* + 1}, and *D*_3_ = {(*b* + 1) < *γθ*}. Note additionally that there is no region defined that corresponds to the equation containing no fixed points. This can be shown either through direct analysis of (16) at steady state or by showing that requiring *b*/*γ* > *θ* and (*b* + 1)/*γ* < *θ* leads to a contradiction. We ignore the hypersurfaces *b* = *γθ* and *b* + 1 = *γθ* as they take up zero volume in $R_{+}^{3}$. This analysis is summarized in Fig 3C, which shows all realizable combinations of fixed point distributions.

At this point, the decomposition framework yields the exact relationship between distribution of fixed points and clearly defined parameter space decomposition. Therefore, given some set of parameters $\left(b,\theta ,\gamma \right)\in R_{+}^{3}$, it is possible to identify which region the parameter set belongs to, as well as identify all adjacent regions that may be traversed through varying parameters. In general, identifying adjacencies between regions requires ideas from computational algebraic geometry, (See S1 Text for details). However, for this simple example, it is clear how these regions must be connected based on the parametrically defined boundaries separating them. The adjacency graph for (16) is provided in Fig 3D, while the divided parameter space is shown in Fig 3E, projected onto (*θ*, *γ*) space.

The primary advantage of analyzing the sharp switch limit of the dynamics is the ability to systematically obtain an explicit algebraic decomposition that separates the entire parameter space into a finite number of regions. However, this is an abstraction from the original problem given in Eq 15. The exact decomposition of parameter space based on the sharp switch limit assists in identifying regions of interest in the finite model by allowing for better pruning of prospective parameter sets. For example, using the defined regions from this sharp switch limit as a guide, we obtained 1000 parameter sets through uniform sampling over (*b*, *γ*, *θ*) ∈ [0.01, 3]^3^ within each region for finite Hill exponent, *n* = 5, 10, and 20. For each parameter set, we then used gradient descent over a range of initial conditions to determine whether the steady state configuration matches the distribution from the Heaviside case, implying that the parameter set is in the same region of parameter space as it was drawn from under the Heaviside limit.

The parameter decomposition based on the dynamics in the Heaviside limit helps guide analysis in the original problem. For example, the proportions in Table 3 show that as *n* decreases, the region of bistability, *D*_2_, is shrinking. This insight demonstrates that parameter sets with the property *b* < *γθ* < *b* + 1 are necessary but not sufficient to induce asymmetry in this simple example system. This same two-step approach is applied to our model in Eqs (11)–(14), enabling us to identify parameter regions that are more dense in the symmetry-breaking steady states needed for oocyte specification in the original, finite model.

**Table 3:** Proportion of parameter sets that remain in the same region under finite Hill dynamics.

Region	*n* = 5	*n* = 10	*n* = 20
**1**	1.0	1.0	1.0
**2**	0.078	0.221	0.449
**3**	1.0	1.0	1.0

### Algebraic decomposition framework

**Fig 4:**
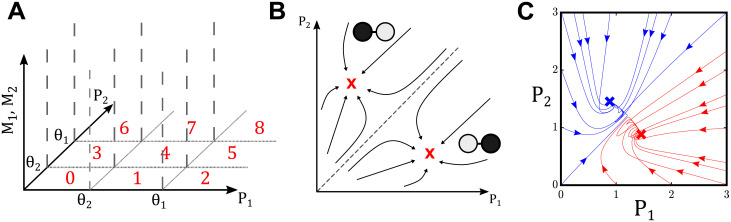
Model decomposition for oocyte selection. (A) Division of *P*_1_, *P*_2_ space into 9 regions based on the values of *θ*_1_ and *θ*_2_. For the Heaviside case, each of the 9 regions defines a distinct set of 4 linear ODEs that can be solved at steady state, yielding a fixed point which may or may not lie within the region. (B) Schematic of desired phase plane dynamics on *P*_1_ and *P*_2_ for the system given by Eqs (11)–(14), where asymmetric steady states (shown in red) will result in the selection of one cell as the oocyte. (C) Example of dynamics that yields only asymmetric steady states over a range of initial conditions. For (*θ*_1_, *θ*_2_, *μ*, *γ*, *η*, *κ*, *π*, *ϵ*) = (1.4, 0.9, 0.8, 0.2, 2.3, 2.5, 1.2, 0.1) with finite Hill exponents *n* = *ν* = 10, a number of initial conditions relax to select cell 1 as the oocyte (red trajectories), while the rest will select cell 2 (shown in blue).

To analyze the model presented in Eqs (11)–(14), and represented by the schematic in Fig 2C, we must identify all possible behaviors this system can produce, paying special attention to the ones that yield symmetry-broken steady states. To do this, we first consider the limit case *n*, *ν* → ∞, where these Hill nonlinearities become Heaviside functions. This simplification changes the system of nonlinear equations into a more complicated combinatorial model, but one that can still be analyzed through our decomposition framework. Here, we analyze the problem for the case *θ*_2_ < *θ*_1_, and we provide results for the case *θ*_1_ < *θ*_2_ in S1 Text. With this inequality, the phase space can be divided into 9 regions, as shown in Fig 4A. For example, the 0th region is defined as the set $\left\{\left(M_{1},M_{2},P_{1},P_{2}\right)\in R_{+}^{4}|P_{1}<\theta_{2},P_{2}<\theta_{2}\right\}$. In this way, each region can be defined as a different case for each Heaviside function taking the value 0 or 1. Within each region, the system of equations is linear.

The linear dynamical system in each region can be shown to have a stable steady state. Let *f*_*i*_, *i* = 0, …, 8 denote the stable fixed point for the dynamics of the system defined by the *i*-th region. One may show that region *i* is attracting for the dynamics of the full system if and only if *f*_*i*_ is in region *i*. Therefore, identifying all possible distributions of attractors in the sharp switch limit is equivalent to analyzing the distribution of *f*_*i*_, *i* = 0, …, 8 over the 9 regions, which can be derived algebraically case-wise. For example, it can be shown when *θ*_2_ < *θ*_1_, *f*_0_ is in the 0th region if and only if 1 < *μπθ*_2_. Similar algebraic restrictions with respect to model parameters can be made for each of the other 8 fixed points relative to their respective regions and for the case where *θ*_1_ < *θ*_2_ (see Tables A and B in S1 Text).

With these definitions, we are able to decompose parameter space into regions corresponding to all possible steady state configurations in the sharp switch limit. After this decomposition, we can sample over each identified region when the sharp switch approximation is relaxed, in search of parameter sets that yield symmetry breaking within our original model. As indicated earlier, the fact that stable fixed points are identified with attracting regions suggests that the dynamics should be robustly preserved under this relaxation. A schematic of the dynamics we seek is shown in Fig 4B. The existence of such dynamics is shown in Fig 4C, where a parameter set was readily found that results in only asymmetric steady states, implying that oocyte selection in the model can be realized for at least one parameter set. We now show how one can map the entire parameter space of a given model with switch-like behavior and how this process can be used to more effectively probe parameter space in search of parameter sets that admit only asymmetric steady states in the finite formulation.

## Results

### Parameter space topology in the sharp switch limit

**Fig 5:**
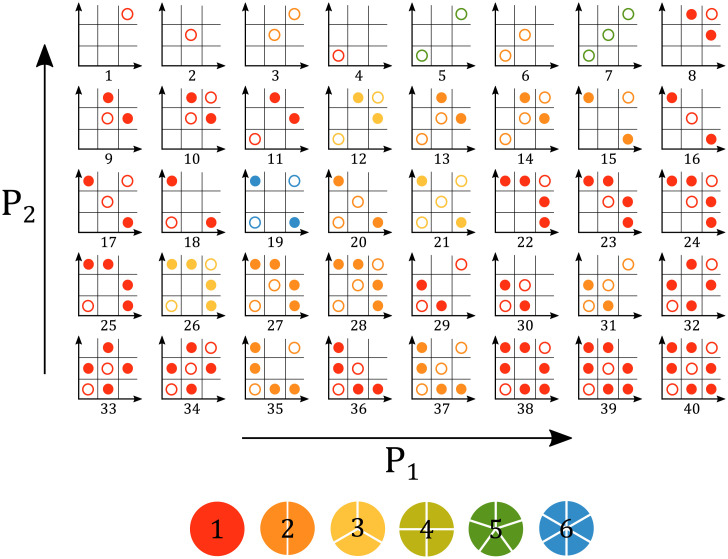
Distribution of stable steady states in the sharp switch limit. Each distinct parameter region is defined by its distribution of attractors within the 9 regions of *P*_1_, *P*_2_ space (from Fig 4), with symmetric steady states denoted by hollow dots and asymmetric steady states denoted as filled dots. The color represents the number of disjoint connected components that exist throughout parameter space that contain the same steady state configuration. For the corresponding distribution in the case *θ*_1_ < *θ*_2_, see Figure B in S1 Text.

After computing the parametric relationships that must hold in each region to contain a fixed point, the parameter space defined by $\left(\theta_{1},\theta_{2},\mu ,\gamma ,\kappa ,\eta ,\pi ,\epsilon \right)\in R_{+}^{8}$ can be divided based on the combination of fixed-point containing regions. That is, each region shown in Fig 5 represents a nonempty region such that parameter sets within this region satisfy some intersection of parametric relationships defined by having some combination of *f*_*i*_ lie within region *i* in *P*_1_, *P*_2_ space. Once divided, it is possible to analyze the topology of all parameter regions and their adjacencies in 8-dimensional parameter space, providing insights into the robustness of symmetry breaking in our model.

**Fig 6:**
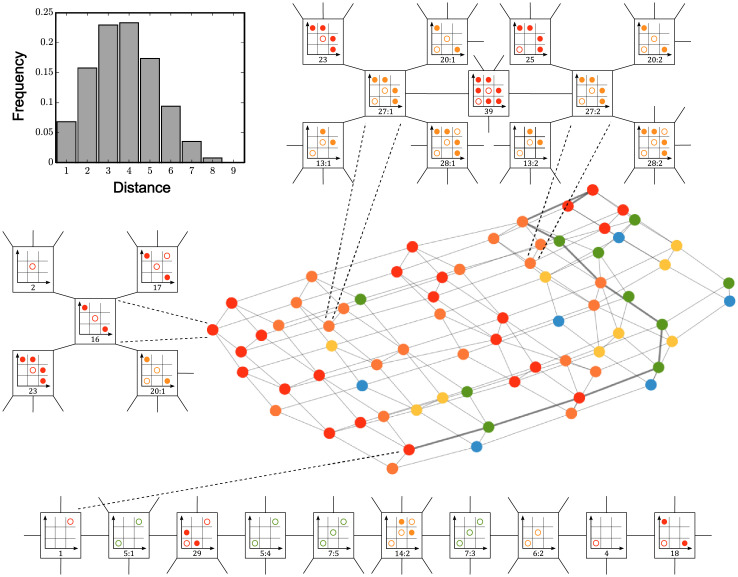
Graph adjacency and topology of parameter space. Adjacency of all regions identified through the division of the entire parameter space under the sharp switch limit. Each node is colored based on how many distinct components exist for each defined region. Top right: inset for adjacency graph between the two components of region 27, along with steady state configurations of each adjacent region. Bottom: inset showing an example path for the diameter of the adjacency graph, along with the steady state configurations of each region along the path. Left: inset for adjacency graph for the singly-defined component of region 16, along with steady state configurations of each adjacent region. Top left: histogram of number of regions separating any pair of nodes in the graph. To see the individual labels for each region, see Figure A in S1 Text. For the corresponding parameter space graph in the case *θ*_1_ < *θ*_2_, see Figure C in S1 Text.

For the simplified Heaviside form of Eqs (11)–(14) with *θ*_2_ < *θ*_1_, we can divide the 8-dimensional parameter space into 70 regions, corresponding to 40 distinct steady state configurations. While some of these steady state configurations exist as a singly-defined region in the parameter space $R_{+}^{8}$, others exist as separate, disjoint regions based on their respective parametric definitions. The distribution of stable steady states within each distinct region is shown in Fig 5, while the adjacencies of all regions in the full parameter space are shown in Fig 6.

In addition to steady state behavior and adjacency within parameter space, each region is explicitly defined by the derived parametric relationships that must hold to realize its characterizing steady state configuration. Although these relationships tend to be rather complex, they may provide a glimpse into the most important characteristics necessary for desirable behaviors within the system. For example, Region 16 is defined as containing all parameter sets (*θ*_1_, *θ*_2_, *μ*, *γ*, *η*, *κ*, *π*, *ϵ*) that satisfy the following inequalities:

$$\begin{array}{c} \frac{\left(1+\gamma \right)\left(1+\eta \right)\left(2+\mu \right)\epsilon +(2+\mu (1+\kappa ))\left(\pi +\epsilon \right)}{\left(\pi +2\epsilon \right)((1+\mu )(1+\kappa )+1)}<\pi \mu \theta_{2}<1, \end{array}$$

$$\begin{array}{c} 1<\frac{\left(1+\gamma )(1+\eta \right)}{1+\kappa }<\pi \mu \theta_{1}<\frac{\left(1+\gamma \right)\left(1+\eta \right)\left(2+\mu \right)\left(\pi +\epsilon \right)+(2+\mu (1+\kappa ))\epsilon }{\left(\pi +2\epsilon \right)((1+\mu )(1+\kappa )+1)}. \end{array}$$

These inequalities provide some insights about basic parametric relationships that may serve as guidelines when searching for global parametric properties that are highly selected in symmetric breaking states. For example, for a parameter set to be in Region 16, we expect that the product *πμ* is sufficiently small, such that it satisfies *πμθ*_2_ < 1. Since *π* and *μ* are related to timescales of protein and mRNA degradation, respectively, to mRNA transport, a small product implies low degradation rates (or conversely high mRNA transport) within the system. If this is true, however, these inequalities also imply that either the threshold for mRNA transport inhibition, *θ*_1_, must be large, or similarly that the rate of protein translation must be large. While there is no governing law for how to select for symmetry breaking within the system, these heuristics provide useful insights into understanding the deeply complex division of parameter space.

The remaining parametric relationships that define each region of parameter space are provided in S1 Text. These boundaries also define the hypersurfaces that separate adjacent regions. That is, in the entire parameter space, these relationships also define the codimension-1 bifurcations that separate distinct regions in the entire eight-dimensional parameter space.

With the successful decomposition of parameter space and identification of each region’s adjacencies, we may further analyze the otherwise opaque topology and structure dictating all potential steady state behaviors of the system within the sharp switch limit of our model. For example, knowing the adjacencies of all regions allows for a definition of distance based on phenotype to be introduced. For any pair of regions in our decomposed parameter space, the minimum number of regions, *N*, that must be traversed to get from one to the other can be computed. Therefore, the distance between any two regions is given by *d* = *N* + 1. A frequency table showing this property is given in Fig 6. Notably, for any pair of randomly chosen regions in parameter space, the probability that the distance between them is four or less is greater than two-thirds. This key fact simply highlights the high degree of interconnectedness between the regions in our eight dimensional parameter space.

The diameter of this graph may also be computed, defined as the greatest distance between any two regions of parameter space. This maximal distance can be shown to be 9, and one such path with this distance is given in the bottom of Fig 6. In this path, the single symmetric steady state of high protein concentration characteristic of Region 1 crosses into Region 5:1, where it gains a symmetric steady state with low protein concentration. This path then crosses into Region 29, gains a pair of asymmetric steady states, and promptly loses them upon traversing into Region 5:4. Upon continuing to Region 7:5, the system again gains another symmetric steady state, gains another pair of asymmetric steady states in Region 14:2, and then loses them once in Region 7:3. The system then loses its high protein symmetric state in Region 6:2, then its middle symmetric state in Region 4, before finally ending in Region 18, where it gains a pair of asymmetric steady states corresponding to high protein concentration in one cell and low concentration in the other. Together with the distance metric, the diameter demonstrates how different behaviors lie with respect to one another in the full eight dimensional parameter space.

Overall, this framework enables all steady state configurations to be defined, along with their respective parametric relationships in the limit of nonlinear regulatory interactions becoming switch-like. Analysis of the adjacency graph for the decomposition of parameter space provides important insights into how different regions of parameter space are related topologically. On the other hand, understanding these definitions and behaviors in the sharp switch limit is an important step in analyzing which areas of parameter space correspond to a higher density of symmetry-broken states when *n* and *ν* are finite.

### Computational analysis for Hill nonlinearities

Using the defined parameter regions from the Heaviside case as a guide, we may sample parameter sets within a region and identify the locations of stable steady states in *P*_1_, *P*_2_ space, checking whether each fixed point *f*_*i*_ remains in region *i* after this perturbation. In addition, we may also use our parameter space decomposition as a guide to identify regions that are denser, on average, with the behaviors we seek. We can use the density of symmetry breaking behavior, given by the proportion of symmetry breaking parameter sets drawn under uniform sampling, as a rough measure of the robustness of symmetry breaking in a given region of our parameter space.

Uniform sampling from the range [0.01, 3]^8^ to generate a set of 10000 parameter sets, we found only 6 of them permitted purely asymmetric steady state solutions in the finite Hill regime (*n* = *ν* = 10) after long time integration from a wide range of initial conditions. In contrast, when this same procedure was performed for 1000 randomly generated parameter sets from Region 16 within the same range, 323 of them were found to contain only asymmetric states. Under this uniform sampling procedure, this seems to indicate that Region 16 is denser in symmetry breaking behavior than the full parameter space.

The substitution of finite Hill equations into the piecewise linear system of differential equations based on the Heaviside approximation has a number of effects on the defined regions in parameter space [32]. For instance, the sharp switch limit yields no parameter sets that admit only symmetry-broken states, as each space allowed for the existence of at least one symmetric steady state. However in the finite Hill regime, for instance when *n* = *ν* = 10, we find many examples of parameter sets that yield only asymmetric steady states. As each region was parametrically defined from the sharp switch limit, knowing which regions contain non-negligible proportions of parameter sets that yield desired solutions helps identify key features required for robust symmetry breaking.

## Discussion

Animal oocytes start their development within clusters of interconnected cells that exchange molecules and organelles [2–4, 7, 8, 11]. The process of oocyte selection within such clusters is one of the key unresolved questions in oogenesis [9, 13, 14]. Recent studies in *Drosophila* revealed that oocyte selection is foreshadowed by autoregulated accumulation of oocyte-specific mRNA and proteins [16, 19, 20, 23, 33]. Even the simplest models of molecular and cellular processes in early stages of animal oogenesis contain a large number of parameters. This presents a considerable challenge for the systematic mapping of parameter regions corresponding to robust symmetry breaking that is needed for oocyte specification. We have shown how this challenge can be successfully addressed for models with sharp switch nonlinearities by combining topological analysis, symbolic computation, and numerical simulations.

The distribution of possible steady states in the sharp switch limit can be broken down into parametrically-defined relationships, which permit the division of parameter space into regions based on these steady state configurations. Thus, the entire parameter space can be more easily defined based on these parametric relationships. Once these regions are defined, it is possible to perform more refined parameter sampling within each region in search of a high density of some behavior of interest, in this case symmetry breaking. As opposed to more common approaches of parametric analysis in nonlinear dynamical systems, our process is neither statistically driven nor based in singularity theory [34–40].

A key feature of our model of *Drosophila* oocyte specification is the existence of asymmetric, stable steady states for the case of symmetric transport between the two central cells in the developing cluster. The presence of these states suggests that, independent of the initial amounts of *orb* mRNA and Orb protein within these two central cells during development, oocyte selection would be guaranteed given a permissible parameter set. This finding is consistent with studies of *orb* mutant cysts, where it was shown that altering an untranslated region of *orb* had a temporal effect on the oocyte selection process, but ultimately did not appear to affect the development of cysts that specified an oocyte. Additionally, it was shown that removing this untranslated region in the mRNA prevented Orb autoregulatory interactions, ultimately resulting in failure for these cysts to properly specify an oocyte [20]. In this way, our model agrees with the idea that selection of the oocyte does not require finely tuned parameter values and that the nonlinear interactions of Orb protein with *orb* mRNA are key in driving the symmetry breaking of the cyst. That is, based on the autoregulatory behavior of Orb protein in the two central cells, our model provides evidence that a symmetric, self-organizing system is sufficient to explain robust oocyte specification.

Analytically, our model is simple and we are seeking simple dynamics. The challenge arises because the parameter space is eight dimensional and classical methods in nonlinear dynamics are not designed to handle high dimensional parameter spaces. We made use of the analytical framework that is based on identifying attracting regions. For our case, the attracting regions of interest are in one-to-one correspondence with the stable equilibria of the Heaviside model, and the associated algebraic conditions are used to obtain an explicit algebraic decomposition of parameter space where the dynamics of each region is constant (See S1 Text for details). For similar models without transport terms, this decomposition is readily available via the open source DSGRN software package [28] that can easily handle systems with higher dimensional parameter spaces.

The fundamental decomposition theorem of Conley [41] guarantees that any dynamical system can be globally decomposed using attracting regions. As a consequence, the DSGRN software can be used to search for a broad range of dynamical features including oscillatory phenomena. Furthermore, attracting regions are closely related to Lyapunov functions [41–43] which are commonly used to provide robust descriptions of global dynamics. Thus, it is not surprising that identification of dynamics in a sharp switch limit gives strong intuition of the behavior of the dynamics for models with finite Hill exponents. The utility of the procedure we have developed comes from the ability to identify regions of interest based on the decomposition of parameter space. Thus, the exact solutions from the sharp switch limit help guide the search for behaviors of interest in finite models. The most cumbersome aspect of the DSGRN approach is that the descriptions of parameter regions—even though, or perhaps because, they are exact—consist of long lists of inequalities and can be difficult to comprehend. However, the hope is that further analysis of these regions, individually and via the adjacency relations, combined with biological insights, may lead to a greater understanding of how parameters influence the emergent dynamic phenotypes.

## Materials and methods

### Fluorescence *in situ* Hybridization (FISH)

Oligonucleotide probes for orb were ordered from LGC Biosearch Technologies and coupled to Atto NHS 633 dye (Sigma) and purified using HPLC. The procedure for the FISH experiments follows that of [20]. Ovaries were dissected in PBS and fixed for 30 minutes in 4% paraformaldehyde (Electron Microscopy Services), rinsed in PBS-Tween (0.1% Tween-20) and dehydrated through a series of methanol washes, stored at -20 degrees for 10 minutes in 100% methanol before bring rehydrated into PBS-Tween. Ovaries were then rinsed with PBS-Tween and incubated in wash buffer (4x SSC, 35% formamide, 0.1% Tween) for 15 minutes at 37 degrees. Samples were incubated with oligoFISH probes overnight at 37 degrees in hybridization buffer (10% dextran sulfate, 0.01% salmon sperm ssDNA, 1% vanadyl ribonucleosidase, 0.2% BSA, 4xSSC, 0.1% Tween-20 and 35% formamide). The following day, samples were washed two times in wash buffer at 37 degrees for one hour and mounted to slides in Aqua-Poly/Mount.

### Computational analysis

Tools and methods underlying the DSGRN framework were used to enumerate the regions of parameter space in the Heaviside limit of the model [28]. Enumeration of realizable regions of parameter space was performed using Mathematica. Finite Hill analysis was performed with numerical time integration functions using MATLAB and Python.

## Supporting information

S1 Text Analysis of model equations and algebraic decomposition for parameter space topology. **Figure A in S1 Text**. **Adjacency graphs of parameter space for *θ*_2_ < *θ*_1_**. Connectivity of all regions identified through the division of the entire parameter space under the sharp switch limit. **Figure B in S1 Text**. **Distribution of steady states in the sharp switch limit for *θ*_1_ < *θ*_2_**. Each distinct parameter region is defined by its distribution of attractors within the 9 regions of *P*_1_, *P*_2_ space, with symmetric steady states denoted by hollow dots and asymmetric steady states denoted as filled dots. The color represents the number of disjoint connected components that exist throughout parameter space that contain the same steady state configuration. **Figure C in S1 Text**. **Adjacency graph of parameter space for *θ*_1_ < *θ*_2_**. Connectivity of all regions identified through the division of the entire parameter space under the sharp switch limit. **Table A in S1 Text**. **Parametric inequalities defining regions of *P*_1_, *P*_2_ space for the *θ*_2_ < *θ*_1_ case**. The relationships between *P*_1_, *P*_2_ and *θ*_1_, *θ*_2_ define the values for each Heaviside expression for each steady state in each region. Here, by symmetry, the inequalities defined by conjugate spatial regions (1 & 3, 2 & 6, and 5 & 7) are equivalent. **Table B in S1 Text**. **Parametric inequalities defining regions of *P*_1_, *P*_2_ space for the *θ*_1_ < *θ*_2_ case**. The relationships between *P*_1_, *P*_2_ and *θ*_1_, *θ*_2_ define the values for each Heaviside expression for each steady state in each region. Here, by symmetry, the inequalities defined by conjugate spatial regions (1 & 3, 2 & 6, and 5 & 7) are equivalent.
(PDF)
