# Supplementary material for: Mapping parameter spaces of biological switches
Source: PLoS Comput Biol. 2021 Feb 8;17(2):e1008711. doi: 10.1371/journal.pcbi.1008711 (PMC7895388; doi:10.1371/journal.pcbi.1008711)
Supplement: S1 Text — Figure A in S1 Text. Adjacency graphs of parameter space for θ2 < θ1. Connectivity of all regions identified through the division of the entire parameter space under the sharp switch limit. Figure B in S1 Text. Distribution of steady states in the sharp switch limit for θ1 < θ2. Each distinct parameter region is defined by its distribution of attractors within the 9 regions of P1, P2 space, with symmetric steady states denoted by hollow dots and asymmetric steady states denoted as filled dots. The color represents the number of disjoint connected components that exist throughout parameter space that contain the same steady state configuration. Figure C in S1 Text. Adjacency graph of parameter space for θ1 < θ2. Connectivity of all regions identified through the division of the entire parameter space under the sharp switch limit. Table A in S1 Text. Parametric inequalities defining regions of P1, P2 space for the θ2 < θ1 case. The relationships between P1, P2 and θ1, θ2 define the values for each Heaviside expression for each steady state in each region. Here, by symmetry, the inequalities defined by conjugate spatial regions (1 & 3, 2 & 6, and 5 & 7) are equivalent. Table B in S1 Text. Parametric inequalities defining regions of P1, P2 space for the θ1 < θ2 case. The relationships between P1, P2 and θ1, θ2 define the values for each Heaviside expression for each steady state in each region. Here, by symmetry, the inequalities defined by conjugate spatial regions (1 & 3, 2 & 6, and 5 & 7) are equivalent. (PDF) [file pcbi.1008711.s001.pdf]

Supplementary Information: Mapping parameter spaces of biological switches

Sharp switch limit steady state analysis

In the following section, we analyze the long-term dynamics of the unbiased system in the sharp switch limit, where we replace the Hill nonlinearities by switch-like Heaviside functions, outlining the general process of the Dynamic Signatures Generated by Regulatory Networks (DSGRN) framework [1, 2]. Under the Heaviside approximation, all nonlinear terms become piecewise linear, yielding a non-homogeneous piecewise linear system of differential equations:

$$\begin{pmatrix} \dot{M}_1 \\ \dot{M}_2 \\ \dot{P}_1 \\ \dot{P}_2 \end{pmatrix} = \begin{pmatrix} 1 \\ 1 \\ 0 \\ 0 \end{pmatrix} + \begin{pmatrix} -\mu - \frac{1}{1+\kappa H(P_1-\theta_1)} & \frac{1}{1+\kappa H(P_2-\theta_1)} & 0 & 0 \\ \frac{1}{1+\kappa H(P_1-\theta_1)} & -\mu - \frac{1}{1+\kappa H(P_2-\theta_1)} & 0 & 0 \\ (1+\gamma H(P_1-\theta_2))\left(\frac{1+\eta H(P_1-\theta_1)}{1+\kappa H(P_1-\theta_1)}\right) & 0 & -\pi-\epsilon & \epsilon \\ 0 & (1+\gamma H(P_2-\theta_2))\left(\frac{1+\eta H(P_2-\theta_1)}{1+\kappa H(P_2-\theta_1)}\right) & \epsilon & -\pi-\epsilon \end{pmatrix} \begin{pmatrix} M_1 \\ M_2 \\ P_1 \\ P_2 \end{pmatrix}. \quad (1)$$

In searching for steady states, we divide P_1, P_2 space into 9 regions based on how these coordinates compare with the values of θ_1 and θ_2 (see Figure 3B in the main text). Therefore, within each of the nine regions, the dynamics of this model is linear, as the Heaviside terms are either 0 or 1. Symbolic computations within each region allows for the general description of the steady state for each coordinate, where the Heaviside terms are replaced by 0 or 1 depending on the region:

$$\begin{aligned}
M_1^* &= \frac{(1+\kappa H(P_1-\theta_1)\,(\mu\,(1+\kappa H(P_2-\theta_1)+2)}{\mu\,(\mu\,(1+\kappa H(P_1-\theta_1))\,(1+\kappa H(P_2-\theta_1))+(1+\kappa H(P_1-\theta_1))+(1+\kappa H(P_2-\theta_1)))}, \\
M_2^* &= \frac{(1+\kappa H(P_2-\theta_1)\,(\mu\,(1+\kappa H(P_1-\theta_1)+2)}{\mu\,(\mu\,(1+\kappa H(P_1-\theta_1))\,(1+\kappa H(P_2-\theta_1))+(1+\kappa H(P_1-\theta_1))+(1+\kappa H(P_2-\theta_1)))}, \\
P_1^* &= \frac{(1+\gamma H(P_1-\theta_2))\,(1+\eta H(P_1-\theta_1))\,(\mu\,(1+\kappa H(P_2-\theta_1)+2)\,(\pi+\epsilon)+(1+\gamma H(P_2-\theta_2))\,(1+\eta H(P_2-\theta_1))\,(\mu\,(1+\kappa H(P_1-\theta_1)+2)\,\epsilon}{\pi\mu\,(\pi+2\epsilon)\,(\mu\,(1+\kappa H(P_1-\theta_1))\,(1+\kappa H(P_2-\theta_1))+(1+\kappa H(P_1-\theta_1))+(1+\kappa H(P_2-\theta_1)))}, \\
P_2^* &= \frac{(1+\gamma H(P_1-\theta_2))\,(1+\eta H(P_1-\theta_1))\,(\mu\,(1+\kappa H(P_2-\theta_1)+2)\,\epsilon+(1+\gamma H(P_2-\theta_2))\,(1+\eta H(P_2-\theta_1))\,(\mu\,(1+\kappa H(P_1-\theta_1)+2)\,(\pi+\epsilon)}{\pi\mu\,(\pi+2\epsilon)\,(\mu\,(1+\kappa H(P_1-\theta_1))\,(1+\kappa H(P_2-\theta_1))+(1+\kappa H(P_1-\theta_1))+(1+\kappa H(P_2-\theta_1)))}.
\end{aligned} \quad (2)$$

As we do not consider points at the boundary between the threshold θ values (that is, $P_1, P_2 \neq \theta_1, \theta_2$), we can check the stability of each fixed point using the Jacobian, which takes the same form as the matrix in (1):

$$\mathbf{J} = \begin{pmatrix} -\mu - \frac{1}{1+\kappa H(P_1-\theta_1)} & \frac{1}{1+\kappa H(P_2-\theta_1)} & 0 & 0 \\ \frac{1}{1+\kappa H(P_1-\theta_1)} & -\mu - \frac{1}{1+\kappa H(P_2-\theta_1)} & 0 & 0 \\ (1+\gamma H(P_1-\theta_2))\left(\frac{1+\eta H(P_1-\theta_1)}{1+\kappa H(P_1-\theta_1)}\right) & 0 & -\pi-\epsilon & \epsilon \\ 0 & (1+\gamma H(P_2-\theta_2))\left(\frac{1+\eta H(P_2-\theta_1)}{1+\kappa H(P_2-\theta_1)}\right) & \epsilon & -\pi-\epsilon \end{pmatrix},$$

which has eigenvalues $\lambda = \left\{-\mu, -\pi, -(\pi+2\epsilon), -\left(\frac{1}{1+\kappa H(P_1-\theta_1)} + \frac{1}{1+\kappa H(P_2-\theta_1)} + \mu\right)\right\}$ that are all necessarily negative. This implies that any steady state identified in this sharp switch limit must be stable.

Denote by f_i the stable steady state vector $(M^*_{1,i}, M^*_{2,i}, P^*_{1,i}, P^*_{2,i})$ for the dynamics over region i defined in Figure 3A of the main text. Here, the goal is to find all possible distribution of stable steady states not lying on the boundary, defined by the hyperplanes $P_i = \theta_j$ for $i, j = 1, 2$, and the corresponding parameter regions for each of the distribution of stable steady states. With this constraints, we may claim there is a stable steady state in the i-th region if and only if the point f_i is contained in the i-th region itself. In general, the bounds for region i to contain a stable steady state can be found case-wise using the equations defined in (2). These constraints are given in Table A for the case where $\theta_2 < \theta_1$ and Table B for $\theta_1 < \theta_2$.

For each region i, this leaves a corresponding parametrically defined region $R_i = R_i(\theta_1, \theta_2, \mu, \gamma, \kappa, \eta, \pi, \epsilon)$ in parameter space, whose bounds are given in Tables A and B, corresponding to the parameter values for which the region i contains a stable steady state. A steady state configuration distributed in these nine regions is considered valid if the parametrically defined regions of the corresponding stable steady states have a nonempty intersection.

By symmetries in (2) (see also Tables A and B), we have conjugate regions for which the same parametric inequalities hold, such that a steady state in one of the regions implies there must exist a steady state in its conjugate. These conjugate pairs are: 1 and 3, 2 and 6, 5 and 7. This means that we only need to consider 6 regions in the P_1, P_2 space to determine all valid steady state configurations. Without loss of generality, let us consider the regions R_i, for $i \in N = \{0, 1, 2, 4, 5, 8\}$, corresponding to the lower triangular portion of the P_1, P_2 space: $\{(P_1, P_2) | \; P_1 \geq P_2\}$. Our next goal is to find all valid steady state configurations.

Algebraic decomposition

As previously established by symmetry, three of the nine regions in the P_1, P_2 space do not provide any additional bounds. Therefore, we only consider the regions R_i for $i \in N = \{0, 1, 2, 4, 5, 8\}$ to identify all possible combinations of steady state distributions. Denote the boundary of each region R_i by $B_i = \partial R_i$, $i \in N$. Define $\Xi := \{(\theta_1, \theta_2, \mu, \gamma, \kappa, \eta, \pi, \epsilon) \in \mathbb{R}^8_+\}$ as the full parameter space. For $\Xi' \subset \Xi$, the parameter space without considering the parametrically-defined boundaries from Tables A and B, we can decompose each region into its component parts and analyze their intersections to understand which steady state combinations are possible. This can be enumerated the following way:

$$\Xi' = \Xi \setminus \bigcup_{i \in N} B_i = \bigcap_{i \in N} (\Xi \setminus B_i) \tag{3}$$

Here, we give a detailed decomposition, obtained via symbolic computations in Mathematica 10, of $\Xi \setminus B_i$ based on the definitions of R_i, $i \in N$, for the case where $\theta_2 < \theta_1$ given in Table A. The decomposition for the case $\theta_1 < \theta_2$ can be derived analogously. In addition, defining the following rational functions greatly simplifies the presentation of the decomposition:

$$\begin{aligned}
r_0 &= \frac{1}{\pi\mu}, \\
r_1 &= \frac{(1+\gamma)(\pi+\epsilon)+\epsilon}{\pi\mu(\pi+2\epsilon)}, \\
r_2 &= \frac{(1+\gamma)\epsilon+(\pi+\epsilon)}{\pi\mu(\pi+2\epsilon)}, \\
r_3 &= \frac{(1+\gamma)(1+\eta)(2+\mu)(\pi+\epsilon)+(2+\mu(1+\kappa))\,\epsilon}{\pi\mu(\pi+2\epsilon)\left((1+\mu)(1+\kappa)+1\right)}, \\
r_4 &= \frac{(1+\gamma)(1+\eta)(2+\mu)\epsilon+(2+\mu(1+\kappa))\,(\pi+\epsilon)}{\pi\mu(\pi+2\epsilon)\left((1+\mu)(1+\kappa)+1\right)}, \\
r_5 &= \frac{1+\gamma}{\pi\mu}, \\
r_6 &= \frac{(1+\gamma)(1+\eta)(2+\mu)(\pi+\epsilon)+(1+\gamma)\,(2+\mu(1+\kappa))\,\epsilon}{\pi\mu(\pi+2\epsilon)\left((1+\mu)(1+\kappa)+1\right)}, \\
r_7 &= \frac{(1+\gamma)(1+\eta)(2+\mu)\epsilon+(1+\gamma)\,(2+\mu(1+\kappa))\,(\pi+\epsilon)}{\pi\mu(\pi+2\epsilon)\left((1+\mu)(1+\kappa)+1\right)}, \\
r_8 &= \frac{(1+\gamma)(1+\eta)}{\pi\mu(1+\kappa)}.
\end{aligned} \tag{4}$$

Using these definitions, the decomposition of $\Xi \setminus B_i, i \in N$ can be expressed as:

- **Spatial Region 0**: $\Xi \setminus B_0 = \bigcup_j R_{0,j} = R_{0,1} \cup R_{0,2}$, where $R_{0,1} = \{r_0 < \theta_2\}$ and $R_{0,2} = \{\theta_2 < r_0\}$,
- **Spatial Regions 1 & 3**: $\Xi \setminus B_1 = \bigcup_j R_{1,j} = R_{1,1} \cup R_{1,2} \cup R_{1,3} \cup R_{1,4}$, where $R_{1,1} = \{r_2 < \theta_2\} \cap \{\theta_2 < r_1 < \theta_1\}, R_{1,2} = \{\theta_2 < r_2\}, R_{1,3} = \{r_1 < \theta_2\}$, and $R_{1,4} = \{\theta_1 < r_1\}$,
- **Spatial Regions 2 & 6**: $\Xi \setminus B_2 = \bigcup_j R_{2,j} = R_{2,1} \cup R_{2,2} \cup R_{2,3}$, where $R_{2,1} = \{\theta_1 < r_3\} \cap \{r_4 < \theta_2\}, R_{2,2} = \{r_3 < \theta_1\}$, and $R_{2,3} = \{\theta_2 < r_4\}$,
- **Spatial Region 4**: $\Xi \setminus B_4 = \bigcup_j R_{4,j} = R_{4,1} \cup R_{4,2} \cup R_{4,3}$, where $R_{4,1} = \{\theta_2 < r_5 < \theta_1\}, R_{4,2} = \{r_5 < \theta_2\}$, and $R_{4,3} = \{\theta_1 < r_5\}$,
- **Spatial Regions 5 & 7**: $\Xi \setminus B_5 = \bigcup_j R_{5,j} = R_{5,1} \cup R_{5,2} \cup R_{5,3} \cup R_{5,4}$, where $R_{5,1} = \{\theta_1 < r_6\} \cap \{\theta_2 < r_7 < \theta_1\}, R_{5,2} = \{r_7 < \theta_2\}, R_{5,3} = \{\theta_1 < r_7\}$, and $R_{5,4} = \{r_6 < \theta_1\}$,
- **Spatial Region 8**: $\Xi \setminus B_8 = \bigcup_j R_{8,j} = R_{8,1} \cup R_{8,2}$, where $R_{8,1} = \{r_5 < \theta_1\}$ and $R_{8,2} = \{\theta_1 < r_5\}$.

For convenience we define the components of each region in the following way, leading to a natural definition for covering sets:

$$\begin{aligned}
R'_0 &= \{R_{0,1}, R_{0,2}\} \\
R'_1 &= \{R_{1,1}, R_{1,2}, R_{1,3}, R_{1,4}\} \\
R'_2 &= \{R_{2,1}, R_{2,2}, R_{2,3}\} \\
R'_4 &= \{R_{4,1}, R_{4,2}, R_{4,3}\} \\
R'_5 &= \{R_{5,1}, R_{5,2}, R_{5,3}, R_{5,4}\} \\
R'_8 &= \{R_{8,1}, R_{8,2}\}.
\end{aligned} \tag{5}$$

Definition 1. Given sets $A_i \in R'_i$, for $i \in N$, set $\mathbf{A} := (A_0, A_1, A_2, A_4, A_5, A_8)$. Define the *covering set* of $\mathbf{A}$ to be

$$D(\mathbf{A}) = \bigcap_{i \in N} A_i.$$

If $D(\mathbf{A}) \neq \emptyset$, we call $D(\mathbf{A})$ a *realizable* covering set.

Covering sets have the following useful property which follows directly from their definition:

Proposition 1. *For a given realizable covering set $D(\mathbf{A})$, the distribution of fixed points across the 9 regions must be the same for all parameter values in $D(\mathbf{A})$.*

The total number of covering sets is given by $\prod_{i \in N} |R'_i| = 576$, where $|R'_i|$ is the cardinality of R'_i. In the next section, we show that only 109 of these are realizable covering sets.

Determination of realizable covering set

From the definition of $D(\mathbf{A})$, realizable covering sets are subvarieties and in principle can be computed analytically by algebraic decomposition algorithms, provided $D(\mathbf{A}) \neq \emptyset$. However, in practice these algorithms have doubly exponential complexity, which can easily make these computations unfeasible. So we need a different approach to analyze the realizability of these sets. Our approach is to consider the following problem:

Definition 2. Let S_{11} denote the symmetric group with 11 elements. For an ordered list $\sigma \in S_{11}$, define

$$\Xi_\sigma = \{\xi \in \Xi \mid r_{\sigma(i)}(\xi) < r_{\sigma(i+1)}(\xi), \text{ for } i = 0, \ldots, 10\}$$

where $r_i, i = 0, \ldots, 8$, are defined in (4), and we take θ_1 and θ_2 as the remaining rational functions needed to be ordered.

Now, we wish to find the set $L = \{\sigma \in S_{11} \mid \Xi_\sigma \neq \emptyset\} \subset S_{11}$. For this we apply a useful fact from real algebraic geometry.

Lemma 1. *A variety $V \subset \mathbb{R}^n$ has nonempty interior if and only if $V = \mathbb{R}^n$.*

Proof. We can show this by induction in the dimension n. For $n = 1$, this can be shown directly from the fundamental theorem of algebra. Assume this property holds for $n-1$. Then for n, assume the variety $V \subset \mathbb{R}^n$ contains an interior point p. For any hyperplane H passing through the point p, the set $V \cap H$ can be taken as a variety in $\mathbb{R}^{n-1}$ with an interior point p. From the inductive step, we have $V \cap H = H$. As the above holds for every hyper plane H passing through p, we have $V = \mathbb{R}^n$. $\square$

The following proposition enables us to find the set L and assists in identifying all realizable covering sets.

Proposition 2. *If a covering set $D(\mathbf{A})$ is nonempty, then there exists $\sigma \in L$ such that $\Xi_\sigma \subset D(\mathbf{A})$.*

Proof. First note that $r_0, \dots, r_8, \theta_1, \theta_2$ are distinct rational functions of the parameters. Therefore, from Lemma 1, the varieties $\{\xi \in \Xi \mid r_i(\xi) = r_j(\xi)\}$ for $i \neq j$ have empty interior. Since $D(\mathbf{A})$ is a nonempty open set, we can find $\xi \in D(\mathbf{A})$ such that $r_i(\xi) \neq r_j(\xi)$ for all $i \neq j$. Suppose the ordering of $r_0, \dots, r_8, \theta_1, \theta_2$ at ξ is $r_{k_0}(\xi) < r_{k_1}(\xi) < \cdots < r_{k_{10}}(\xi)$. Then, we can define $\sigma = (k_0, k_2, \dots, k_{10})$ and we have $\Xi_\sigma \neq \emptyset$ since $\xi \in \Xi_\sigma$. Now notice that, if $r_{n_1} < r_{n_2}$ is a defining inequality of $D(\mathbf{A})$, then in particular $r_{n_1}(\xi) < r_{n_2}(\xi)$ which implies that $n_1 = k_i$ and $n_2 = k_{i+j}$, that is, the inequality $r_{n_1}(\xi) < r_{n_2}(\xi)$ is given by the ordering defined by σ. In other words, the inequality $r_{n_1} < r_{n_2}$ is a defining inequality of Ξ_σ. Therefore given $\xi' \in \Xi_\sigma$, it follows that $r_{n_1}(\xi') < r_{n_2}(\xi')$. Since this is true for all inequalities defining $D(\mathbf{A})$ it follows that $\xi' \in D(\mathbf{A})$. Therefore, we have $\Xi_\sigma \subset D(\mathbf{A})$. $\square$

Additionally, for $\sigma \in L$ and a realizable covering set $D(\mathbf{A})$, if $\Xi_\sigma \cap D(\mathbf{A}) \neq \emptyset$, then $\Xi_\sigma \subset D(\mathbf{A})$. The proof of this fact is essentially the same as the second part of the proof of Proposition 2. Moreover, we can show that $D(\mathbf{A})$ can almost be constructed by taking the union of Ξ_σ:

Proposition 3. *Given a realizable covering set $D(\mathbf{A})$, define $S(\mathbf{A}) = \{\sigma \in L \mid \Xi_\sigma \subset D(\mathbf{A})\}$. Then $\mathrm{cl}\,(D(\mathbf{A})) = \cup_{\sigma \in S(\mathbf{A})} \mathrm{cl}\,(\Xi_\sigma)$.*

Proof. Since $\Xi_\sigma \subset D(\mathbf{A})$ for $\sigma \in S(\mathbf{A})$ and $S(\mathbf{A})$ is finite, it follows directly that

$$\bigcup_{\sigma \in S(\mathbf{A})} \mathrm{cl}\,(\Xi_\sigma) \subset \mathrm{cl}\,(D(\mathbf{A}))\,.$$

For the other direction, suppose $\xi \in \mathrm{cl}\,(D(\mathbf{A}))$. Then, there exists a sequence $\{\xi_n\} \subset D(\mathbf{A})$ such that $\xi_n \to \xi$ and, as in the proof of the Proposition 2 above, from Lemma 1 we can choose this sequence such that $\xi_n \in \{\xi \in D(\mathbf{A}) \mid r_i(\xi) \neq r_j(\xi) \text{ for all } i \neq j\}$ for all n. Therefore, since $S(\mathbf{A})$ is finite, there exists $\sigma \in S(\mathbf{A})$ such that infinitely many elements of the sequence belong to Ξ_σ. In other words there exists a subsequence denoted $\{\xi_{n_i}\}$, such that $\{\xi_{n_i}\} \subset \Xi_\sigma$. Hence it follows that $\xi \in \mathrm{cl}\,(\Xi_\sigma)$. $\square$

The following corollary is very useful for the computation of the realizable covering sets.

Corollary 1. *Consider a realizable covering set $D(\mathbf{A})$ and a set Ξ_σ for some $\sigma \in S_{11}$. Let $I_1 = \{(\sigma(i), \sigma(i+1)) \mid i = 0, \dots, 10\}$ and $I_2 = \{(i,j) \mid r_i < r_j$ is a defining inequality of $D(\mathbf{A})\}$. Then we can write $\Xi_\sigma = \{\xi \in \Xi \mid r_i < r_j$ for all $(i,j) \in I_1\}$ and $D(\mathbf{A}) = \{\xi \in \Xi \mid r_i < r_j$ for all $(i,j) \in I_2\}$. Furthermore $\Xi_\sigma \subset D(\mathbf{A})$ if and only if $I_2 \subset I_1$.*

From the results above, once we identify the set L, we can determine whether $D(\mathbf{A})$ is empty or not by checking whether there is a $\sigma \in L$ such that $\Xi_\sigma \subset D(\mathbf{A})$. This step has linear complexity since, by Corollary 1, all we need to do is compare the defining inequalities of $D(\mathbf{A})$ and Ξ_σ.

Definition 3. For $\sigma \in S_9$, define

$$\Xi'_\sigma = \{(\mu, \gamma, \kappa, \eta, \pi, \epsilon) \in (0,\infty)^6 \mid r_{\sigma(i)}(\xi) < r_{\sigma(i+1)}(\xi) \text{ for } i = 0, \dots 8\}$$

where $r_i, i = 0, \dots, 8$ are defined by (4).

Note that θ_1 and θ_2 are values independent of the variables $(\mu, \gamma, \kappa, \eta, \pi, \epsilon)$. Therefore, we can first solve a simpler problem and find the set $L' = \{\sigma \in S_9 \mid \Xi'_\sigma \neq \emptyset\} \subset S_9$ as follows.

In order to find L', we need to check if Ξ'_σ is empty or not for $|S_9| = 9! = 362880$ permutations $\sigma \in S_9$. We implement this search in two steps. In the first step, we use algebraic restrictions on the relationships between the r_i to remove σ values for which Ξ'_σ is empty, leaving a much smaller set $S' \subset S_9$ with $L' \subset S'$. In the second step, we show that $\Xi'_\sigma \neq \emptyset$ for all $\sigma \in S'$ by finding a sample point $\xi'_\sigma \in \Xi'_\sigma$ for each $\sigma \in S'$.

For the first step, r_i, $i = 0, \ldots, 8$, have the following relationships, which can be found by simple algebraic manipulations over $(\mu, \gamma, \kappa, \eta, \pi, \epsilon) \in (0, \infty)^6$. To get these relationships we put all r_i over the same denominator and directly compare each case, using the additional fact that by definition, $\eta < \kappa$. Doing so yields the following restrictions:

$$\begin{array}{llllllll} r_0 < r_1, & r_0 < r_2, & r_0 < r_3, & r_0 < r_5, & r_0 < r_6, & r_0 < r_8, & r_1 > r_2, & r_1 < r_3, \\ r_1 < r_5, & r_1 < r_6, & r_1 < r_8, & r_2 < r_3, & r_2 < r_5, & r_2 < r_6, & r_2 < r_8, & r_3 > r_4, \\ r_3 < r_6, & r_4 < r_6, & r_4 < r_7, & r_4 < r_8, & r_5 < r_6, & r_5 < r_8, & r_6 > r_7, & r_7 < r_8. \end{array}$$

These restrictions greatly reduce the number of possible cases that need to be checked for possible ordering lists given by $\sigma \in S_9$. For example, $r_0 < r_1$ means that to have $\sigma(i) = 0$ and $\sigma(j) = 1$, it is necessary that $i < j$, that is, 0 must appear before 1 in the list σ. Under these restrictions over S_9, the set $S' \subset S_9$ can be generated, with the following $|S'| = 37$ elements:

	$(0,4,2,1,3,7,5,8,6)$		$(0,4,2,1,7,3,5,8,6)$	
$(0,2,4,1,7,5,8,3,6)$	$(0,2,1,5,4,7,8,3,6)$	$(0,2,1,5,4,7,3,6,8)$	$(0,2,1,4,7,5,8,3,6)$	$(0,4,2,1,7,5,8,3,6)$
$(0,2,4,1,5,3,7,6,8)$	$(0,2,1,5,4,7,3,8,6)$	$(0,2,1,4,3,7,5,8,6)$	$(0,2,1,4,5,3,7,6,8)$	$(0,2,4,1,7,3,5,8,6)$
$(0,2,1,4,5,3,7,8,6)$	$(0,2,4,7,1,5,8,3,6)$	$(0,2,4,1,5,3,7,8,6)$	$(0,2,4,1,5,7,3,8,6)$	$(0,4,7,2,1,5,8,3,6)$
$(0,2,1,4,3,5,7,6,8)$	$(0,4,2,7,1,5,8,3,6)$	$(0,2,4,1,3,5,7,6,8)$	$(4,0,7,2,1,5,8,3,6)$	$(0,2,1,4,7,5,3,8,6)$
$(0,2,1,4,5,7,8,3,6)$	$(0,2,4,1,7,5,3,8,6)$	$(0,2,1,4,5,7,3,6,8)$	$(0,2,4,1,3,7,5,8,6)$	$(0,2,1,4,3,5,7,8,6)$
$(0,2,1,5,4,3,7,6,8)$	$(0,2,1,4,5,7,3,8,6)$	$(0,2,4,1,5,7,8,3,6)$	$(4,7,0,2,1,5,8,3,6)$	$(4,0,2,7,1,5,8,3,6)$
$(0,4,2,1,7,5,3,8,6)$	$(0,2,4,1,3,5,7,8,6)$	$(0,2,1,5,4,3,7,8,6)$	$(0,2,4,1,5,7,3,6,8)$	$(0,2,1,4,7,3,5,8,6)$

For the second step, we want to show that there is a sample point $\xi'_\sigma \in \Xi'_\sigma$ for each $\sigma \in S'$. This can be done by using the `FindInstance` function in Mathematica 10. Given a system of equations or inequalities, this function returns a single list of parameter values such that all of the expressions are true, thus proving that the set is nonempty. Calling this function with the inequalities corresponding to each element of S' we get that $\Xi'_\sigma \neq \emptyset$ for all $\sigma \in S'$, and hence that $L' = S'$.

In order to derive the set L from L', we must place the 9 components of each list $\sigma' \in L'$ into the 11 spots of the lists $\sigma \in S_{11}$ to make a list in L. There are $\frac{11!}{9!2!} = 55$ ways to do this. Since in the case we are treating now $(\theta_2 < \theta_1)$, we only have one choice on how to insert these into each list obtained by inserting the elements of a $\sigma' \in L'$ into a list in S_{11}. Therefore we have $|L| = 55$, $|L'| = 2035$ lists in L.

From Proposition 2, Corollary 1, and the resulting discussion, we can find the set of all realizable covering sets, denoted by D, by checking whether the defining inequalities of $D(\mathbf{A})$ is a subset of the defining inequalities of Ξ_σ for some $\sigma \in S_{11}$. Checking this for all 2035 lists in L, we find $|D| = 109$. For convenience we denote the set of all realizable covering sets by $D = \{D_1, \ldots, D_{109}\}$.

Determination of distribution of fixed points

Now that we have obtained the set D, we can find all possible distributions of fixed points over $\Xi' = \bigcup_{i=1}^{109} D_i$, the parameter space without the parametric boundaries. By Proposition 1, the fixed point distribution is constant over each D_i. Therefore, for each $D_i \in D$, we can check whether $R_k \subset D_i$, for $k \in \{0, 1, 2, 4, 5, 8\}$, where R_k are the parameter regions whose bounds are given by Tables A and B, by comparing their defining inequalities. Performing these comparisons, we find 40 different distributions of fixed points that are realizable in the system.

Geometry of realizable covering sets

In this section we provide a method to show how the sets in D are interconnected. Moreover, we present efficient methods for checking whether sets in D, say D_i and D_j, have a nonempty intersection and to check if they are adjacent to each other, with the notion of adjacency to be defined.

Now we can introduce an efficient method to check whether $D_i \cap D_j$ is empty or not. The method follows from the following proposition.

Proposition 4. *For two realizable covering sets D_i and D_j, $D_i \cap D_j \neq \emptyset$ if and only if there exists $\sigma \in L$ such that $\Xi_\sigma \subset D_i$ and $\Xi_\sigma \subset D_j$.*

Proof. The if part is trivial. If there is a $\sigma \in L$ such that $\Xi_\sigma \subset D_i$ and $\Xi_\sigma \subset D_j$, then $\Xi_\sigma \subset D_i \cap D_j$ and hence the intersection is not empty.

For the only if part, suppose $D_i \cap D_j \neq \emptyset$ with $i \neq j$. Since $D_i \cap D_j$ is an open set, proceeding as in the proof of Proposition 2, we can find a $\xi \in D_i \cap D_j$ such that $r_{i_1}(\xi) \neq r_{i_2}(\xi)$ for all $i_1, i_2 = 0, \ldots, 10$ with $i_1 \neq i_2$. Assuming that the order of $r_0, \ldots, r_8, \theta_1, \theta_2$ at ξ is $r_{k_0}(\xi) < r_{k_1}(\xi) < \cdots < r_{k_9}(\xi)$ we can define $\sigma = (k_0, k_1, \ldots, k_{10})$ and have that $\Xi_\sigma \neq \emptyset$ as $\xi \in \Xi_\sigma$. Again, continuing as in the proof of Proposition 2, observe that if $r_{i_1} < r_{i_2}$ is one of the defining inequalities of D_i or D_j then, since $\xi \in \Xi_\sigma$ and $r_{i_1}(\xi) < r_{i_2}(\xi)$, we must have that $r_{i_1} < r_{i_2}$ is one of the defining inequalities of Ξ_σ. Therefore $\Xi_\sigma \subset D_i$ and $\Xi_\sigma \subset D_j$. □

From this result it follows that for each pair D_i, D_j, in order to check if $D_i \cap D_j$ is empty or not, we simply need to check whether there is a $\sigma \in L$ such that the defining inequalities of D_i and D_j are all contained in the set of the defining inequalities of Ξ_σ.

The following proposition, whose proof follows directly from the definition of the realizable covering sets, will be used in the next section in the discussion of adjacency for disjoint decomposition sets.

Proposition 5. *For a realizable covering set D_k, denoting its boundary by $\mathrm{bd}(D_k)$, we have*

$$\mathrm{bd}(D_k) \subset \bigcup_{i \in \{1,2\},\, k \in \{0,\ldots,7\}} \{\theta_i - r_k = 0\}$$

Topology of parameter regions with respect to fixed point distributions

We do not know whether each realizable covering sets $D_i, i = 1, \ldots, 109$ is connected or not, but from Proposition 2 we know whether $D_i \cap D_j, i, j = 1, \ldots, 109$ is empty or not. This implies that we can find a decomposition of the set of parameters corresponding to a giving fixed point distribution by checking, via Proposition 2, whether any two of its realizable coverings D_i and D_j have empty intersection or not. The distinct regions and their decomposition for the case $\theta_2 < \theta_1$ are tabulated below, where for convenience we use S_1, S_2, and S_3 to represent the elements in D. In a slight abuse of notation, each set S_j is presented as a 4-tuple $(S_j^1, S_j^2, S_j^3, S_j^4)$ representing the set

$$S_j = \{\xi \in \Xi \mid \max_{i \in I_1(S_j)} r_i = r_{j_1} < \theta_1 < \min_{i \in I_2(S_j)} r_i = r_{j_2} \text{ and } \max_{i \in I_3(S_j)} r_i = r_{j_3} < \theta_2 < \min_{i \in I_4(S_j)} r_i = r_{j_4}\},$$

where $r_{j_i} \in S_j^i$ for $i = 1, \ldots, 4$. In some cases, the S_j^i have multiple elements, indicating that the minimum or maximum of these rational functions may change depending on the parameter values. Blank entries for S_j^i in S_j imply that the corresponding θ_1 or θ_2 is unbounded in that direction.

For the case $\theta_2 < \theta_1$, there are 40 distinct regions with 70 disjoint decomposition sets, listed as follows:

- **Parameter Region 1**: $C_1 = S_1 \cup S_2 \cup S_3$, where $S_1 = ((), (r_7, r_5), (), (r_4, r_0))$, $S_2 = ((), (r_1, r_7), (), (r_4, r_0))$, $S_3 = ((r_3,), (r_7, r_5), (), (r_0,))$
- **Parameter Region 2**: $C_2 = S_1 \cup S_2$, where $S_1 = ((r_8, r_3), (), (r_7,), (r_0,))$, $S_2 = ((r_8, r_6), (), (), (r_0,))$
- **Parameter Region 3:1**: $C_3 = S_1$, where $S_1 = ((r_6,), (r_8,), (), (r_0,))$
- **Parameter Region 3:2**: $C_4 = S_1 \cup S_2$, where $S_1 = ((r_5,), (r_7,), (), (r_4, r_0))$, $S_2 = ((r_5, r_3), (r_7,), (), (r_0,))$

- **Parameter Region 4**: $C_5 = S_1 \cup S_2$, where $S_1 = ((r_8, r_3), (), (r_7, r_5), ())$, $S_2 = ((r_8, r_6), (), (r_5,), ())$
- **Parameter Region 5:1**: $C_6 = S_1 \cup S_2 \cup S_3$, where $S_1 = ((), (r_7, r_5), (r_0,), (r_2, r_4))$, $S_2 = ((), (r_1, r_7), (r_0,), (r_4,))$, $S_3 = ((r_3,), (r_7, r_5), (r_0,), (r_2,))$
- **Parameter Region 5:2**: $C_7 = S_1$, where $S_1 = ((r_3,), (r_5,), (r_1, r_7), ())$
- **Parameter Region 5:3**: $C_8 = S_1 \cup S_2$, where $S_1 = ((r_3,), (r_8,), (r_7, r_5), ())$, $S_2 = ((r_6,), (r_8,), (r_5,), ())$
- **Parameter Region 5:4**: $C_9 = S_1 \cup S_2$, where $S_1 = ((), (r_7, r_5), (r_1,), (r_4,))$, $S_2 = ((r_3,), (r_7, r_5), (r_1,), ())$
- **Parameter Region 5:5**: $C_{10} = S_1 \cup S_2$, where $S_1 = ((), (r_7,), (r_5,), (r_4,))$, $S_2 = ((r_3,), (r_7,), (r_5,), ())$
- **Parameter Region 6:1**: $C_{11} = S_1 \cup S_2$, where $S_1 = ((r_8, r_3), (), (r_7, r_0), (r_2,))$, $S_2 = ((r_8, r_6), (), (r_0,), (r_2,))$
- **Parameter Region 6:2**: $C_{12} = S_1 \cup S_2$, where $S_1 = ((r_8, r_3), (), (r_1, r_7), (r_5,))$, $S_2 = ((r_8, r_6), (), (r_1,), (r_5,))$
- **Parameter Region 7:1**: $C_{13} = S_1$, where $S_1 = ((r_6,), (r_8,), (r_0,), (r_2,))$
- **Parameter Region 7:2**: $C_{14} = S_1 \cup S_2$, where $S_1 = ((r_5,), (r_7,), (r_0,), (r_2, r_4))$, $S_2 = ((r_5, r_3), (r_7,), (r_0,), (r_2,))$
- **Parameter Region 7:3**: $C_{15} = S_1$, where $S_1 = ((r_6,), (r_8,), (r_1,), (r_5,))$
- **Parameter Region 7:4**: $C_{16} = S_1$, where $S_1 = ((r_5, r_3), (r_8,), (r_1, r_7), (r_5,))$
- **Parameter Region 7:5**: $C_{17} = S_1 \cup S_2$, where $S_1 = ((r_5,), (r_7,), (r_1,), (r_4, r_5))$, $S_2 = ((r_5, r_3), (r_7,), (r_1,), (r_5,))$
- **Parameter Region 8**: $C_{18} = S_1 \cup S_2 \cup S_3$, where $S_1 = ((r_7,), (r_5,), (), (r_4, r_0))$, $S_2 = ((r_7,), (r_1,), (), (r_4, r_0))$, $S_3 = ((r_7, r_3), (r_5,), (), (r_7, r_0))$
- **Parameter Region 9**: $C_{19} = S_1 \cup S_2$, where $S_1 = ((r_8,), (r_6,), (), (r_4, r_0))$, $S_2 = ((r_8, r_3), (r_6,), (), (r_7, r_0))$
- **Parameter Region 10**: $C_{20} = S_1 \cup S_2$, where $S_1 = ((r_7, r_5), (r_8, r_6), (), (r_4, r_0))$, $S_2 = ((r_7, r_5, r_3), (r_8, r_6), (), (r_7, r_0))$
- **Parameter Region 11**: $C_{21} = S_1 \cup S_2$, where $S_1 = ((r_8,), (r_6,), (r_5,), (r_4,))$, $S_2 = ((r_8, r_3), (r_6,), (r_5,), (r_7,))$
- **Parameter Region 12:1**: $C_{22} = S_1 \cup S_2 \cup S_3$, where $S_1 = ((r_7,), (r_5,), (r_0,), (r_2, r_4))$, $S_2 = ((r_7,), (r_1,), (r_0,), (r_4,))$, $S_3 = ((r_7, r_3), (r_5,), (r_0,), (r_2, r_7))$
- **Parameter Region 12:2**: $C_{23} = S_1 \cup S_2$, where $S_1 = ((r_7,), (r_5,), (r_1,), (r_4,))$, $S_2 = ((r_7, r_3), (r_5,), (r_1,), (r_7,))$
- **Parameter Region 12:3**: $C_{24} = S_1 \cup S_2$, where $S_1 = ((r_7,), (r_8, r_6), (r_5,), (r_4,))$, $S_2 = ((r_7, r_3), (r_8, r_6), (r_5,), (r_7,))$
- **Parameter Region 13:1**: $C_{25} = S_1 \cup S_2$, where $S_1 = ((r_8,), (r_6,), (r_0,), (r_2, r_4))$, $S_2 = ((r_8, r_3), (r_6,), (r_0,), (r_2, r_7))$
- **Parameter Region 13:2**: $C_{26} = S_1 \cup S_2$, where $S_1 = ((r_8,), (r_6,), (r_1,), (r_4, r_5))$, $S_2 = ((r_8, r_3), (r_6,), (r_1,), (r_7, r_5))$
- **Parameter Region 14:1**: $C_{27} = S_1 \cup S_2$, where $S_1 = ((r_7, r_5), (r_8, r_6), (r_0,), (r_2, r_4))$, $S_2 = ((r_7, r_5, r_3), (r_8, r_6), (r_0,), (r_2, r_7))$
- **Parameter Region 14:2**: $C_{28} = S_1 \cup S_2$, where $S_1 = ((r_7, r_5), (r_8, r_6), (r_1,), (r_4, r_5))$, $S_2 = ((r_7, r_5, r_3), (r_8, r_6), (r_1,), (r_7, r_5))$

- **Parameter Region 15:1**: $C_{29} = S_1 \cup S_2$, where $S_1 = ((), (r_7, r_5, r_3), (r_4,), (r_0,))$, $S_2 = ((), (r_1, r_7), (r_4,), (r_0,))$
- **Parameter Region 15:2**: $C_{30} = S_1 \cup S_2$, where $S_1 = ((), (r_5, r_3), (r_7,), (r_0,))$, $S_2 = ((), (r_1,), (r_7,), (r_0,))$
- **Parameter Region 16**: $C_{31} = S_1$, where $S_1 = ((r_8,), (r_3,), (r_7,), (r_0,))$
- **Parameter Region 17**: $C_{32} = S_1$, where $S_1 = ((r_5,), (r_8, r_3), (r_7,), (r_0,))$
- **Parameter Region 18**: $C_{33} = S_1$, where $S_1 = ((r_8,), (r_3,), (r_7, r_5), ())$
- **Parameter Region 19:1**: $C_{34} = S_1 \cup S_2$, where $S_1 = ((), (r_7, r_5, r_3), (r_4, r_0), (r_2,))$, $S_2 = ((), (r_1, r_7), (r_4, r_0), ())$
- **Parameter Region 19:2**: $C_{35} = S_1 \cup S_2$, where $S_1 = ((), (r_5, r_3), (r_7, r_0), (r_2,))$, $S_2 = ((), (r_1,), (r_7, r_0), ())$
- **Parameter Region 19:3**: $C_{36} = S_1$, where $S_1 = ((), (r_7, r_5, r_3), (r_1, r_4), ())$
- **Parameter Region 19:4**: $C_{37} = S_1$, where $S_1 = ((), (r_7, r_3), (r_4, r_5), ())$
- **Parameter Region 19:5**: $C_{38} = S_1$, where $S_1 = ((), (r_5, r_3), (r_1, r_7), ())$
- **Parameter Region 19:6**: $C_{39} = S_1$, where $S_1 = ((), (r_8, r_3), (r_7, r_5), ())$
- **Parameter Region 20:1**: $C_{40} = S_1$, where $S_1 = ((r_8,), (r_3,), (r_7, r_0), (r_2,))$
- **Parameter Region 20:2**: $C_{41} = S_1$, where $S_1 = ((r_8,), (r_3,), (r_1, r_7), (r_5,))$
- **Parameter Region 21:1**: $C_{42} = S_1$, where $S_1 = ((r_5,), (r_8, r_3), (r_7, r_0), (r_2,))$
- **Parameter Region 21:2**: $C_{43} = S_1$, where $S_1 = ((r_5,), (r_7, r_3), (r_1, r_4), (r_5,))$
- **Parameter Region 21:3**: $C_{44} = S_1$, where $S_1 = ((r_5,), (r_8, r_3), (r_1, r_7), (r_5,))$
- **Parameter Region 22**: $C_{45} = S_1 \cup S_2$, where $S_1 = ((r_7,), (r_5, r_3), (r_4,), (r_7, r_0))$, $S_2 = ((r_7,), (r_1,), (r_4,), (r_7, r_0))$
- **Parameter Region 23**: $C_{46} = S_1$, where $S_1 = ((r_8,), (r_3,), (r_4,), (r_7, r_0))$
- **Parameter Region 24**: $C_{47} = S_1$, where $S_1 = ((r_7, r_5), (r_8, r_3), (r_4,), (r_7, r_0))$
- **Parameter Region 25**: $C_{48} = S_1$, where $S_1 = ((r_8,), (r_3,), (r_4, r_5), (r_7,))$
- **Parameter Region 26:1**: $C_{49} = S_1 \cup S_2$, where $S_1 = ((r_7,), (r_5, r_3), (r_4, r_0), (r_2, r_7))$, $S_2 = ((r_7,), (r_1,), (r_4, r_0), (r_7,))$
- **Parameter Region 26:2**: $C_{50} = S_1$, where $S_1 = ((r_7,), (r_5, r_3), (r_1, r_4), (r_7,))$
- **Parameter Region 26:3**: $C_{51} = S_1$, where $S_1 = ((r_7,), (r_8, r_3), (r_4, r_5), (r_7,))$
- **Parameter Region 27:1**: $C_{52} = S_1$, where $S_1 = ((r_8,), (r_3,), (r_4, r_0), (r_2, r_7))$
- **Parameter Region 27:2**: $C_{53} = S_1$, where $S_1 = ((r_8,), (r_3,), (r_1, r_4), (r_7, r_5))$
- **Parameter Region 28:1**: $C_{54} = S_1$, where $S_1 = ((r_7, r_5), (r_8, r_3), (r_4, r_0), (r_2, r_7))$
- **Parameter Region 28:2**: $C_{55} = S_1$, where $S_1 = ((r_7, r_5), (r_8, r_3), (r_1, r_4), (r_7, r_5))$
- **Parameter Region 29**: $C_{56} = S_1 \cup S_2$, where $S_1 = ((r_1,), (r_7, r_5), (r_2,), (r_1, r_4))$, $S_2 = ((r_3,), (r_7, r_5), (r_2,), (r_1,))$
- **Parameter Region 30**: $C_{57} = S_1 \cup S_2$, where $S_1 = ((r_8, r_3), (), (r_2, r_7), (r_1,))$, $S_2 = ((r_6, r_8), (), (r_2,), (r_1,))$

- **Parameter Region 31:1**: $C_{58} = S_1$, where $S_1 = ((r_6,),(r_8,),(r_2,),(r_1,))$
- **Parameter Region 31:2**: $C_{59} = S_1 \cup S_2$, where $S_1 = ((r_5,),(r_7,),(r_2,),(r_1,r_4))$, $S_2 = ((r_5,r_3),(r_7,),(r_2,),(r_1,))$
- **Parameter Region 32**: $C_{60} = S_1 \cup S_2$, where $S_1 = ((r_1,r_7),(r_5,),(r_2,),(r_1,r_4))$, $S_2 = ((r_7,r_3),(r_5,),(r_2,),(r_1,r_7))$
- **Parameter Region 33**: $C_{61} = S_1 \cup S_2$, where $S_1 = ((r_8,),(r_6,),(r_2,),(r_1,r_4))$, $S_2 = ((r_3,r_8),(r_6,),(r_2,),(r_1,r_7))$
- **Parameter Region 34**: $C_{62} = S_1 \cup S_2$, where $S_1 = ((r_7,r_5),(r_8,r_6),(r_2,),(r_1,r_4))$, $S_2 = ((r_7,r_5,r_3),(r_8,r_6),(r_2,),(r_1,r_7))$
- **Parameter Region 35:1**: $C_{63} = S_1$, where $S_1 = ((r_1,),(r_7,r_5,r_3),(r_2,r_4),(r_1,))$
- **Parameter Region 35:2**: $C_{64} = S_1$, where $S_1 = ((r_1,),(r_5,r_3),(r_2,r_7),(r_1,))$
- **Parameter Region 36**: $C_{65} = S_1$, where $S_1 = ((r_8,),(r_3,),(r_2,r_7),(r_1,))$
- **Parameter Region 37:1**: $C_{66} = S_1$, where $S_1 = ((r_5,),(r_7,r_3),(r_2,r_4),(r_1,))$
- **Parameter Region 37:2**: $C_{67} = S_1$, where $S_1 = ((r_5,),(r_8,r_3),(r_2,r_7),(r_1,))$
- **Parameter Region 38**: $C_{68} = S_1$, where $S_1 = ((r_1,r_7),(r_5,r_3),(r_2,r_4),(r_1,r_7))$
- **Parameter Region 39**: $C_{69} = S_1$, where $S_1 = ((r_8,),(r_3,),(r_2,r_4),(r_1,r_7))$
- **Parameter Region 40**: $C_{70} = S_1$, where $S_1 = ((r_7,r_5),(r_8,r_3),(r_2,r_4),(r_1,r_7))$

For the case $\theta_1 < \theta_2$, there are 38 distinct regions, with 69 disjoint decomposition sets. In this case, we define the additional rational functions r_9 through r_{13} as follows:

$$
\begin{aligned}
r_9 &= \frac{(1+\eta)(2+\mu)(\pi+\epsilon)+(2+\mu)\epsilon}{\pi\mu(\pi+2\epsilon)\left((1+\mu)(1+\kappa)+1\right)},\\
r_{10} &= \frac{(1+\eta)(2+\mu)\epsilon+(2+\mu)(\pi+\epsilon)}{\pi\mu(\pi+2\epsilon)\left((1+\mu)(1+\kappa)+1\right)},\\
r_{11} &= \frac{(1+\gamma)}{\pi\mu},\\
r_{12} &= \frac{(1+\gamma)(1+\eta)(\pi+\epsilon)+(1+\eta)\epsilon}{\pi\mu(\pi+2\epsilon)(1+\kappa)},\\
r_{13} &= \frac{(1+\gamma)(1+\eta)\epsilon+(1+\eta)(\pi+\epsilon)}{\pi\mu(\pi+2\epsilon)(1+\kappa)}.
\end{aligned}
\tag{6}
$$

The parameter regions and their disjoint decomposition sets are listed below:

- **Parameter Region 1**: $C_1 = S_1 \cup S_2$, where $S_1 = ((),(r_0,r_4),(),(r_9,r_{11}))$, $S_2 = ((),(r_0,r_{10}),(),(r_{11},))$
- **Parameter Region 2**: $C_2 = S_1 \cup S_2$, where $S_1 = ((),(r_0,r_4),(r_8,),(r_9,))$, $S_2 = ((),(r_0,r_{10}),(r_8,),())$
- **Parameter Region 3:1**: $C_3 = S_1 \cup S_2$, where $S_1 = ((),(r_0,r_4),(r_{12},),(r_9,r_8))$, $S_2 = ((),(r_0,r_{10}),(r_{12},),(r_8,))$
- **Parameter Region 3:2**: $C_4 = S_1 \cup S_2$, where $S_1 = ((),(r_0,r_4),(r_{11},),(r_9,r_{13}))$, $S_2 = ((),(r_0,r_{10}),(r_{11},),(r_{13},))$

- **Parameter Region 4**: $C_5 = S_1 \cup S_2 \cup S_3 \cup S_4$, where $S_1 = ((r_9, r_{11}), (), (r_3, r_8), ())$, $S_2 = ((r_9, r_{13}), (), (r_3, r_8), ())$, $S_3 = ((r_9, r_{11}), (r_4,), (r_8,), ())$, $S_4 = ((r_9, r_{13}), (r_4,), (r_8,), ())$
- **Parameter Region 5:1**: $C_6 = S_1 \cup S_2 \cup S_3 \cup S_4$, where $S_1 = ((r_9, r_{11}), (), (r_3, r_{12}), (r_8,))$, $S_2 = ((r_9, r_{13}), (), (r_3,), (r_8,))$, $S_3 = ((r_9, r_{11}), (r_4,), (r_{12},), (r_8,))$, $S_4 = ((r_9, r_{13}), (r_4,), (), (r_8,))$
- **Parameter Region 5:2**: $C_7 = S_1 \cup S_2$, where $S_1 = ((r_9, r_{11}), (), (r_3,), (r_{13},))$, $S_2 = ((r_9, r_{11}), (r_4,), (), (r_{13},))$
- **Parameter Region 5:3**: $C_8 = S_1 \cup S_2$, where $S_1 = ((r_9,), (), (r_3,), (r_{11},))$, $S_2 = ((r_9,), (r_4,), (), (r_{11},))$
- **Parameter Region 5:4**: $C_9 = S_1$, where $S_1 = ((r_{11},), (r_4,), (r_{12},), (r_9, r_8))$
- **Parameter Region 5:5**: $C_{10} = S_1$, where $S_1 = ((r_{11},), (r_4,), (), (r_9, r_{13}))$
- **Parameter Region 5:6**: $C_{11} = S_1 \cup S_2$, where $S_1 = ((r_0,), (r_4,), (), (r_9, r_{11}))$, $S_2 = ((r_0,), (r_{10},), (), (r_{11},))$
- **Parameter Region 6:1**: $C_{12} = S_1 \cup S_2$, where $S_1 = ((r_9,), (r_{11},), (r_3, r_8), ())$, $S_2 = ((r_9,), (r_{11}, r_4), (r_8,), ())$
- **Parameter Region 6:2**: $C_{13} = S_1 \cup S_2$, where $S_1 = ((r_0,), (r_{11}, r_4), (r_8,), (r_9,))$, $S_2 = ((r_0,), (r_{10},), (r_8,), ())$
- **Parameter Region 7:1**: $C_{14} = S_1 \cup S_2$, where $S_1 = ((r_9,), (r_{11},), (r_3, r_{12}), (r_8,))$, $S_2 = ((r_9,), (r_{11}, r_4), (r_{12},), (r_8,))$
- **Parameter Region 7:2**: $C_{15} = S_1 \cup S_2$, where $S_1 = ((r_9,), (r_{11},), (r_3, r_{11}), (r_{13},))$, $S_2 = ((r_9,), (r_{11}, r_4), (r_{11},), (r_{13},))$
- **Parameter Region 7:3**: $C_{16} = S_1 \cup S_2$, where $S_1 = ((r_0,), (r_{11}, r_4), (r_{12},), (r_9, r_8))$, $S_2 = ((r_0,), (r_{10},), (r_{12},), (r_8,))$
- **Parameter Region 7:4**: $C_{17} = S_1 \cup S_2$, where $S_1 = ((r_0,), (r_{11}, r_4), (r_{11},), (r_9, r_{13}))$, $S_2 = ((r_0,), (r_{10},), (r_{11},), (r_{13},))$
- **Parameter Region 8**: $C_{18} = S_1 \cup S_2$, where $S_1 = ((), (r_0, r_4), (r_{13},), (r_9, r_{12}))$, $S_2 = ((), (r_{10}, r_0), (r_{13},), (r_{12},))$
- **Parameter Region 9:1**: $C_{19} = S_1 \cup S_2$, where $S_1 = ((r_9, r_{11}), (r_{13},), (r_3, r_{13}), (r_{12},))$, $S_2 = ((r_9, r_{11}), (r_{13}, r_4), (r_{13},), (r_{12},))$
- **Parameter Region 9:2**: $C_{20} = S_1$, where $S_1 = ((r_{11},), (r_{13}, r_4), (r_{13},), (r_9, r_{12}))$
- **Parameter Region 10:1**: $C_{21} = S_1 \cup S_2$, where $S_1 = ((r_9,), (r_{11},), (r_3, r_{13}), (r_{12},))$, $S_2 = ((r_9,), (r_{11}, r_4), (r_{13},), (r_{12},))$
- **Parameter Region 10:2**: $C_{22} = S_1 \cup S_2$, where $S_1 = ((r_0,), (r_{11}, r_4), (r_{13},), (r_9, r_{12}))$, $S_2 = ((r_0,), (r_{10},), (r_{13},), (r_{12},))$
- **Parameter Region 11**: $C_{23} = S_1$, where $S_1 = ((r_4,), (r_0,), (), (r_9, r_{11}))$
- **Parameter Region 12**: $C_{24} = S_1$, where $S_1 = ((r_4,), (r_0,), (r_8,), (r_9,))$
- **Parameter Region 13:1**: $C_{25} = S_1$, where $S_1 = ((r_4,), (r_0,), (r_{12},), (r_9, r_8))$
- **Parameter Region 13:2**: $C_{26} = S_1$, where $S_1 = ((r_4,), (r_0,), (r_{11},), (r_9, r_{13}))$
- **Parameter Region 14:1**: $C_{27} = S_1 \cup S_2$, where $S_1 = ((r_9, r_{11}, r_4), (), (r_8,), (r_3,))$, $S_2 = ((r_9, r_{13}, r_4), (), (r_8,), (r_3,))$
- **Parameter Region 14:2**: $C_{28} = S_1 \cup S_2$, where $S_1 = ((r_{11}, r_4), (), (r_8,), (r_9,))$, $S_2 = ((r_{13}, r_4), (), (r_8,), (r_9,))$
- **Parameter Region 15:1**: $C_{29} = S_1 \cup S_2$, where $S_1 = ((r_9, r_{11}, r_4), (), (r_{12},), (r_3, r_8))$, $S_2 = ((r_9, r_{13}, r_4), (), (), (r_3, r_8))$

- **Parameter Region 15:2**: $C_{30} = S_1$, where $S_1 = ((r_9, r_{11}, r_4), (), (), (r_3, r_{13}))$
- **Parameter Region 15:3**: $C_{31} = S_1$, where $S_1 = ((r_9, r_4), (), (), (r_3, r_{11}))$
- **Parameter Region 15:4**: $C_{32} = S_1 \cup S_2$, where $S_1 = ((r_{11}, r_4), (), (r_{12},), (r_9, r_8))$, $S_2 = ((r_{13}, r_4), (), (), (r_9, r_8))$
- **Parameter Region 15:5**: $C_{33} = S_1$, where $S_1 = ((r_{11}, r_4), (), (), (r_9, r_{13}))$
- **Parameter Region 15:6**: $C_{34} = S_1$, where $S_1 = ((r_0, r_4), (), (), (r_9, r_{11}))$
- **Parameter Region 16**: $C_{35} = S_1$, where $S_1 = ((r_0, r_4), (r_{11},), (r_8,), (r_9,))$
- **Parameter Region 17:1**: $C_{36} = S_1$, where $S_1 = ((r_9, r_4), (r_{11},), (r_{12},), (r_3, r_8))$
- **Parameter Region 17:2**: $C_{37} = S_1$, where $S_1 = ((r_9, r_4), (r_{11},), (r_{11},), (r_3, r_{13}))$
- **Parameter Region 17:3**: $C_{38} = S_1$, where $S_1 = ((r_0, r_4), (r_{11},), (r_{12},), (r_9, r_8))$
- **Parameter Region 17:4**: $C_{39} = S_1$, where $S_1 = ((r_0, r_4), (r_{11},), (r_{11},), (r_9, r_{13}))$
- **Parameter Region 18**: $C_{40} = S_1$, where $S_1 = ((r_4,), (r_0,), (r_{13},), (r_9, r_{12}))$
- **Parameter Region 19:1**: $C_{41} = S_1$, where $S_1 = ((r_9, r_{11}, r_4), (r_{13},), (r_{13},), (r_3, r_{12}))$
- **Parameter Region 19:2**: $C_{42} = S_1$, where $S_1 = ((r_{11}, r_4), (r_{13},), (r_{13},), (r_9, r_{12}))$
- **Parameter Region 20:1**: $C_{43} = S_1$, where $S_1 = ((r_9, r_4), (r_{11},), (r_{13},), (r_3, r_{12}))$
- **Parameter Region 20:2**: $C_{44} = S_1$, where $S_1 = ((r_0, r_4), (r_{11},), (r_{13},), (r_9, r_{12}))$
- **Parameter Region 21**: $C_{45} = S_1 \cup S_2$, where $S_1 = ((r_{10},), (r_0,), (r_3, r_8), ())$, $S_2 = ((r_{10},), (r_0, r_4), (r_9, r_8), ())$
- **Parameter Region 22:1**: $C_{46} = S_1$, where $S_1 = ((r_{10},), (r_0, r_4), (r_9, r_{11}), (r_{13},))$
- **Parameter Region 22:2**: $C_{47} = S_1 \cup S_2$, where $S_1 = ((r_{10},), (r_0,), (r_3, r_{12}), (r_8,))$, $S_2 = ((r_{10},), (r_0, r_4), (r_9, r_{12}), (r_8,))$
- **Parameter Region 23**: $C_{48} = S_1 \cup S_2 \cup S_3$, where $S_1 = ((r_{11},), (r_9,), (r_3, r_8), ())$, $S_2 = ((r_{11},), (r_9, r_4), (r_9, r_8), ())$, $S_3 = ((r_{13},), (r_9,), (r_3, r_8), ())$
- **Parameter Region 24:1**: $C_{49} = S_1$, where $S_1 = ((r_{11},), (r_9, r_4), (r_9,), (r_{13},))$
- **Parameter Region 24:2**: $C_{50} = S_1 \cup S_2$, where $S_1 = ((r_{11},), (r_9,), (r_3, r_{12}), (r_8,))$, $S_2 = ((r_{11},), (r_9, r_4), (r_9, r_{12}), (r_8,))$
- **Parameter Region 24:3**: $C_{51} = S_1 \cup S_2$, where $S_1 = ((r_0, r_{10}), (r_9,), (r_3,), (r_{11},))$, $S_2 = ((r_0, r_{10}), (r_9, r_4), (r_9,), (r_{11},))$
- **Parameter Region 25**: $C_{52} = S_1 \cup S_2$, where $S_1 = ((r_0, r_{10}), (r_9, r_{11}), (r_3, r_8), ())$, $S_2 = ((r_0, r_{10}), (r_9, r_{11}, r_4), (r_9, r_8), ())$
- **Parameter Region 26:1**: $C_{53} = S_1 \cup S_2$, where $S_1 = ((r_0, r_{10}), (r_9, r_{11}), (r_3, r_{12}), (r_8,))$, $S_2 = ((r_0, r_{10}), (r_9, r_{11}, r_4), (r_9, r_{12}), (r_8,))$
- **Parameter Region 26:2**: $C_{54} = S_1 \cup S_2$, where $S_1 = ((r_0, r_{10}), (r_9, r_{11}), (r_3, r_{11}), (r_{13},))$, $S_2 = ((r_0, r_{10}), (r_9, r_{11}, r_4), (r_9, r_{11}), (r_{13},))$
- **Parameter Region 27**: $C_{55} = S_1$, where $S_1 = ((r_{10},), (r_4, r_0), (r_9, r_{13}), (r_{12},))$
- **Parameter Region 28**: $C_{56} = S_1$, where $S_1 = ((r_{11},), (r_9, r_{13}, r_4), (r_9, r_{13}), (r_{12},))$

- **Parameter Region 29**: $C_{57} = S_1 \cup S_2$, where $S_1 = ((r_0, r_{10}), (r_9, r_{11}), (r_3, r_{13}), (r_{12},))$, $S_2 = ((r_0, r_{10}), (r_9, r_{11}, r_4), (r_9, r_{13}), (r_{12},))$
- **Parameter Region 30**: $C_{58} = S_1$, where $S_1 = ((r_4,), (r_0,), (r_9, r_8), (r_3,))$
- **Parameter Region 31**: $C_{59} = S_1$, where $S_1 = ((r_4,), (r_0,), (r_9, r_{12}), (r_3, r_8))$
- **Parameter Region 32**: $C_{60} = S_1 \cup S_2$, where $S_1 = ((r_{11}, r_4), (r_9,), (r_9, r_8), (r_3,))$, $S_2 = ((r_{13}, r_4), (r_9,), (r_9, r_8), (r_3,))$
- **Parameter Region 33:1**: $C_{61} = S_1 \cup S_2$, where $S_1 = ((r_{11}, r_4), (r_9,), (r_9, r_{12}), (r_3, r_8))$, $S_2 = ((r_{13}, r_4), (r_9,), (r_9,), (r_3, r_8))$
- **Parameter Region 33:2**: $C_{62} = S_1$, where $S_1 = ((r_{11}, r_4), (r_9,), (r_9,), (r_3, r_{13}))$
- **Parameter Region 33:3**: $C_{63} = S_1$, where $S_1 = ((r_0, r_4), (r_9,), (r_9,), (r_3, r_{11}))$
- **Parameter Region 34**: $C_{64} = S_1$, where $S_1 = ((r_0, r_4), (r_9, r_{11}), (r_9, r_8), (r_3,))$
- **Parameter Region 35:1**: $C_{65} = S_1$, where $S_1 = ((r_0, r_4), (r_9, r_{11}), (r_9, r_{12}), (r_3, r_8))$
- **Parameter Region 35:2**: $C_{66} = S_1$, where $S_1 = ((r_0, r_4), (r_9, r_{11}), (r_9, r_{11}), (r_3, r_{13}))$
- **Parameter Region 36**: $C_{67} = S_1$, where $S_1 = ((r_4,), (r_0,), (r_9, r_{13}), (r_3, r_{12}))$
- **Parameter Region 37**: $C_{68} = S_1$, where $S_1 = ((r_{11}, r_4), (r_9, r_{13}), (r_9, r_{13}), (r_3, r_{12}))$
- **Parameter Region 38**: $C_{69} = S_1$, where $S_1 = ((r_0, r_4), (r_9, r_{11}), (r_9, r_{13}), (r_3, r_{12}))$

Adjacency between disjoint sets

We will now present an efficient algorithm to check whether two realizable sets are adjacent or not. For this, we introduce the definition of a regular boundary point and adjacency between two sets. Note that, in order to study adjacency, we need to consider the whole space $\Xi = (0, \infty)^8$, rather than the parameter space without the boundary points between regions $\Xi' = \Xi \setminus \bigcup_{i=1 \in N} B_i$.

We first introduce the Baire category theorem (see [3]), which will be useful in some of the subsequent proofs.

Theorem 1 (Baire category theorem)**.** *In a locally compact Hausdorff space, if V_i, $i = 1, 2, \dots$ are countably many closed sets without interior points, then $\bigcup_{i=1}^{\infty} V_i$ also has no interior points.*

Definition 4. Given two open sets $X, Y \subset \mathbb{R}^n$ with $X \cap Y = \emptyset$ and $\mathrm{cl}(X) \cap \mathrm{cl}(Y) \neq \emptyset$, we say that a point $\xi \in \mathrm{cl}(X) \cap \mathrm{cl}(Y)$ is a *regular boundary point* of X and Y if there exist an $\epsilon > 0$ and a differential function $f \colon B_\epsilon(\xi) \to \mathbb{R}$, with non singular Jacobian, such that $B_\epsilon(\xi) \cap \mathrm{cl}(X) \cap \mathrm{cl}(Y) = \{x \in B_\epsilon(\xi) \mid f(x) = 0\}$, where $B_\epsilon(\xi)$ is the ball of radius ϵ centered at ξ. We say that the sets X and Y are *adjacent* if they admit at least one regular boundary point $\xi \in \mathrm{cl}(X) \cap \mathrm{cl}(Y)$.

If two sets in $\mathbb{R}^n$ are adjacent, the definition above says that their common boundary is locally a $(n-1)$-dimensional regular manifold (hypersurface) given by $\{f = 0\}$. From the definition above, if ξ is a regular boundary point, then any point $\xi' \in B_\epsilon(\xi) \cap \mathrm{cl}(X) \cap \mathrm{cl}(Y)$ is also a regular boundary point. We now prove the following proposition, which will be useful in the later computations:

Proposition 6. *Suppose that X and Y are two open sets and $\{U_i\}_{i \in I}$, $\{V_j\}_{j \in J}$ are two collections of open sets such that $U_i \subset X$ for all $i \in I$, $V_j \subset Y$ for all $j \in J$, $\mathrm{cl}(X) = \bigcup_{i \in I} \mathrm{cl}(U_i)$, $\mathrm{cl}(Y) = \bigcup_{j \in J} \mathrm{cl}(V_j)$ and I and J are finite sets. Then X and Y are adjacent if and only if U_i and V_j are adjacent for some $i \in I$ and some $j \in J$.*

Proof. The if part follows directly from Definition 4. Now, suppose that X and Y are adjacent and f, ξ, and ϵ are as in Definition 4. Since $\mathrm{cl}(X) = \bigcup_{i\in I} \mathrm{cl}(U_i)$ and $\mathrm{cl}(Y) = \cup_{j\in J} \mathrm{cl}(V_j)$, it follows that $B_\epsilon(\xi) \cap \{f = 0\} = B_\epsilon(\xi) \cap \bigcup_{i\in I, j\in J} \mathrm{cl}(U_i) \cap \mathrm{cl}(V_j)$. From the Baire category theorem, we get that there exist $i \in I$ and $j \in J$ such that $B_\epsilon(\xi) \cap \mathrm{cl}(U_i) \cap \mathrm{cl}(V_j)$ has an interior point ξ' with respect to the relative topology of $B_\epsilon(\xi) \cap \{f = 0\}$. In other words, there exists an $\epsilon' > 0$ such that $B_{\epsilon'}(\xi') \cap \{f = 0\} = B_{\epsilon'}(\xi') \cap \mathrm{cl}(U_i) \cap \mathrm{cl}(V_j)$. Furthermore, since $U_i \subset X$ and $V_j \subset Y$ and X and Y are adjacent, and hence disjoint, we have $U_i \cap V_j = \emptyset$. This shows that U_i and V_j are adjacent and that ξ' is a regular boundary point of U_i and V_j. □

The following corollary follows directly form Proposition 3 and Proposition 6.

Corollary 2. *Any two realizable covering sets D_i and D_j are adjacent if and only if there exist $\sigma_1, \sigma_2 \in L$ such that $\Xi_{\sigma_1} \subset D_i$, $\Xi_{\sigma_2} \subset D_j$, and Ξ_{σ_1} and Ξ_{σ_2} are adjacent.*

Going one step further, from the construction of the disconnected decomposition sets, we know that each disconnected decomposition sets is a finite union of realizable covering set, so Propositions 3 and 6, imply the following:

Proposition 7. *Any two disjoint decomposition sets C_i and C_j are adjacent if and only if there exist $\sigma_1, \sigma_2 \in L$ such that $\Xi_{\sigma_1} \subset C_i$, $\Xi_{\sigma_2} \subset C_j$, and Ξ_{σ_1} and Ξ_{σ_2} are adjacent.*

We now define the notions of adjacency and θ-adjacency for permutations $\sigma \in S_{11}$, that will be used in the sequence.

Definition 5. Given $\sigma_1, \sigma_2 \in S_{11}$, we say that they are *adjacent* if and only if there exists some $m \in \{0, \dots, 9\}$ such that $\sigma_1(j) = \sigma_2(j)$ for all $j \in \{0, \dots, 10\} \setminus \{m, m+1\}$ and $\sigma_1(m) = \sigma_2(m+1)$ and $\sigma_1(m+1) = \sigma_2(m)$. Furthermore, we say σ_1 and σ_2 are *θ-adjacent* if they are adjacent and $\{\sigma_1(m), \sigma_1(m+1)\} \cap \{9, 10\} \neq \emptyset$ and $\{\sigma_1(m), \sigma_1(m+1)\} \cap \{0, \dots, 8\} \neq \emptyset$.

The interpretation of the notion of adjacency in the definition above is that σ_1 and σ_2 are adjacent if and only if the defining inequalities of Ξ_{σ_1} and Ξ_{σ_2} differ by switching exactly one inequality, and they are θ-adjacent if they differ by switching exactly one inequality between a θ_i and a $r_j \notin \{\theta_1, \theta_2\}$. This definition allows us to directly prove the following propositions. The first one, whose proof is based on algebra of permutations and is left to reader, is basically a rewrite of the definition of adjacency and says that if σ_1 and σ_2 are not adjacent then at least two defining inequalities of Ξ_{σ_1} and Ξ_{σ_2} are switched, and that if they are not θ-adjacent then either they are not adjacent or the switched inequality does not involve θ_i.

Proposition 8. *Let $\sigma_1 \neq \sigma_2 \in S_{11}$. If σ_1 and σ_2 are not adjacent, then there exists i, j, k, l, with $(i, j) \neq (k, l)$, such that $\sigma_1^{-1}(i) < \sigma_1^{-1}(j)$, $\sigma_2^{-1}(i) > \sigma_2^{-1}(j)$, $\sigma_1^{-1}(k) < \sigma_1^{-1}(l)$, and $\sigma_2^{-1}(k) > \sigma_2^{-1}(l)$. If σ_1 and σ_2 are not θ-adjacent, then they are either not adjacent or $\sigma_1^{-1}(i) < \sigma_1^{-1}(j)$ and $\sigma_2^{-1}(i) > \sigma_2^{-1}(j)$ for some $i, j \in \{0, \dots, 8\}$ with $|i - j| = 1$.*

Proposition 9. *If $i, j \in \{0, \dots, 10\}$ and $\sigma_1, \sigma_2 \in L$ are such that $\sigma_1^{-1}(i) < \sigma_1^{-1}(j)$ and $\sigma_2^{-1}(i) > \sigma_2^{-1}(j)$, then $\mathrm{cl}(\Xi_{\sigma_1}) \cap \mathrm{cl}(\Xi_{\sigma_2}) \subset \{r_i - r_j = 0\}$.*

Proof. From their definition we have that $\Xi_{\sigma_1} \subset \{r_i < r_j\}$ and $\Xi_{\sigma_2} \subset \{r_j < r_i\}$. It then follows that $\mathrm{cl}(\Xi_{\sigma_1}) \cap \mathrm{cl}(\Xi_{\sigma_2}) \subset \{r_i \leq r_j\} \cap \{r_i \geq r_j\} = \{r_i = r_j\}$. □

Moving forward, we have the following proposition, whose proof is obtained by computing the Jacobian and its rank in Mathematica 10:

Proposition 10. *Let $i, j, k, l \in \{0, \dots, 10\}$ such that $i \neq j$, $k \neq l$, $(i, j) \neq (k, l)$, $(i, j) \neq (l, k)$, and $\{i, j, k, l\} \cap \{9, 10\} \neq \emptyset$. Then the Jacobian of the vector valued function $(r_i - r_j, r_k - r_l)$ has rank 2 over Ξ. In other words, $\{r_i - r_j = 0, r_k - r_l = 0\}$ defines a regular $(n-2)$-dimensional manifold.*

Proof. As $\{\theta_i - r_k = 0, \theta_i - r_l = 0\} = \{\theta_i - r_l = 0, r_k - r_l = 0\}$, we only need to consider cases, $\{\theta_i - r_l = 0, r_k - r_l = 0\}$ and $\{\theta_i - r_l = 0, \theta_i - r_l = 0\}$. It is easy to see the rank of vector valued function $(\theta_i - r_k, \theta_i - r_l)$ is 2 as $i \neq j$. For case $\{\theta_i - r_l = 0, r_k - r_l = 0\}$, we use Mathematica 10 to show $\partial_{\mu,\gamma,\kappa,\eta,\pi,\epsilon}(r_k - r_j) \neq 0$ which indicates the rank of the vector valued function is 2. □

Here, we have shown that the varieties $\{\theta_i - r_k = 0, \theta_i - r_l = 0\}$, $\{\theta_i - r_k = 0, \theta_j - r_l = 0\}$, and $\{\theta_i - r_l = 0, r_k - r_l = 0\}$ are regular $(n-2)$-dimensional manifolds in Ξ. We now provide the final proposition needed to check the adjacency of the sets Ξ_σ:

Proposition 11. *Let D_p and D_q be two disjoint realizable covering sets and $\sigma_1, \sigma_2 \in L$ such that $\Xi_{\sigma_1} \subset D_p$ and $\Xi_{\sigma_2} \in D_q$. Then Ξ_{σ_1} and Ξ_{σ_2} are adjacent if and only if σ_1 and σ_2 are θ-adjacent.*

Proof. If σ_1 and σ_2 are θ-adjacent with $\sigma_1(m) = \sigma_2(m+1)$ and $\sigma_1(m+1) = \sigma_2(m)$, without loss of generality suppose that $\sigma_1(m) = k \in \{0, \ldots, 8\}$ and $\sigma_1(m+1) = 9$. Note that this means that $r_k(\xi) < \theta_1$ for $\xi \in \Xi_{\sigma_1}$ and $r_k(\xi) > \theta_1$ for $\xi \in \Xi_{\sigma_2}$. Then, we can choose a point $\xi = (\lambda, \theta_1) \in \Xi_{\sigma_1}$ and define a new point $\xi' = (\lambda, \theta_1')$, where $\theta_1' = r_k(\lambda)$, to have $\theta_1' = r_k(\lambda) < \theta_1$. It then follows that there exists $\epsilon > 0$, such that $B_\epsilon(\xi') \cap \{r_k < \theta_1\} = B_\epsilon(\xi') \cap \Xi_{\sigma_1}$, $B_\epsilon(\xi') \cap \{r_k > \theta_1\} = B_\epsilon(\xi') \cap \Xi_{\sigma_2}$, and $B_\epsilon(\xi') \cap \mathrm{cl}(\Xi_{\sigma_1}) \cap \mathrm{cl}(\Xi_{\sigma_2}) = B_\epsilon(\xi') \cap \{r_k - \theta_1 = 0\}$. This implies that Ξ_{σ_1} and Ξ_{σ_2} are adjacent and ξ' is a regular boundary point.

Now suppose that Ξ_{σ_1} and Ξ_{σ_2} are adjacent. If σ_1 and σ_2 are not θ-adjacent, then from Proposition 8 and Proposition 9 we have either $\mathrm{cl}(\Xi_{\sigma_1}) \cap \mathrm{cl}(\Xi_{\sigma_2}) \subset \{r_i - r_j = 0\}$, for some $i, j \in \{0, \ldots, 8\}$ or $\mathrm{cl}(\Xi_{\sigma_1}) \cap \mathrm{cl}(\Xi_{\sigma_2}) \subset \{r_j - \theta_i = 0, r_l - \theta_k = 0\}$ for some $(j, l) \in \{0, \ldots, 8\}$, $(i, k) \in \{1, 2\}$, with $(i, j) \neq (j, l)$. In the first case, if $\mathrm{cl}(\Xi_{\sigma_1}) \cap \mathrm{cl}(\Xi_{\sigma_2}) \subset \{r_{k_0} - r_{l_0} = 0\}$, for some $(k_0, l_0) \in \{0, \ldots, 8\}$, since we have from Proposition 5 that $\mathrm{cl}(D_p) \cap \mathrm{cl}(D_q) \subset \mathrm{bd}(D_p) \cup \mathrm{bd}(D_q) \subset \bigcup_{i \in \{1,2\}, k \in \{0,\ldots,8\}} \{\theta_i - r_k = 0\}$, it follows that $\mathrm{cl}(\Xi_{\sigma_1}) \cap \mathrm{cl}(\Xi_{\sigma_2}) \subset \bigcup_{i \in \{1,2\}, k \in \{0,\ldots,8\}} \{\theta_i - r_k = 0\} \cap \{r_{k_0} - r_{l_0} = 0\}$. Note that, from Proposition 10, it follows that the right hand side of this last inclusion is a finite union of regular $(n-2)$-dimensional manifolds, and hence by Definition 4, we conclude that Ξ_{σ_1} and Ξ_{σ_2} are not adjacent. In the second case, it follows from Proposition 10 that $\{r_j - \theta_i = 0, r_l - \theta_k = 0\}$ is a regular $(n-2)$-dimensional manifold, and therefore that Ξ_{σ_1} and Ξ_{σ_2} are not adjacent. □

Checking the θ-adjacency of σ_1, σ_2 can be done efficiently by checking the order of their defining rational functions r_i, $i \in \{0, \ldots, 10\}$. Using this method we checked the adjacency for all disjoint decomposition sets C_j, $j = 1, \ldots, 70$ for the case $\theta_2 < \theta_1$ and $j = 1, \ldots, 69$ for the case $\theta_1 < \theta_2$. We represent the adjacency relationship between the regions as a graph, where two disjoint decomposition sets C_i and C_j are connected by an edge if and only if they are adjacent.

The adjacency graph for the $\theta_2 < \theta_1$ and $\theta_1 < \theta_2$ cases are shown in Figures A and C, respectively. The corresponding steady state distributions for each distinct region identified for each case are provided in Figure 5 of the main text (for $\theta_2 < \theta_1$) and B (for $\theta_1 < \theta_2$).

Analysis of system with finite Hill coefficients

If the sharp switch limit is not induced, we have the dynamical system represented by Equations (11) – (14) of the main text. The decomposition presented above yields explicit bounds in parameter space and define all possible distributions of steady states in P_1, P_2 space. However, we may use the decomposition of parameter space given from the sharp switch limit as a guide to more effectively sample regions of parameter space that may guarantee symmetry-breaking solutions.

Within each region, parameter sets can be randomly generated from $(\mu, \gamma, \kappa, \eta, \pi, \epsilon, \theta_1, \theta_2) \in [0.01, 3]^8$ subject to the additional parameter constraints within each parameter region at a given ν and n. For each parameter set, each steady state can be identified using gradient descent and its stability checked based on the Jacobian. For each steady state $(M_1^*, M_2^*, P_1^*, P_2^*)$, the Jacobian is given by the following matrix:

$$\mathbf{J} = \begin{pmatrix} -\mu - \frac{1}{1+\kappa f(P_1^*)} & \frac{1}{1+\kappa f(P_2^*)} & \frac{\kappa f'(P_1^*) M_1^*}{(1+\kappa f(P_1^*))^2} & -\frac{\kappa f'(P_2^*) M_2^*}{(1+\kappa f(P_2^*))^2} \\ \frac{1}{1+\kappa f(P_1^*)} & -\mu - \frac{1}{1+\kappa f(P_2^*)} & -\frac{\kappa f'(P_1^*) M_1^*}{(1+\kappa f(P_1^*))^2} & \frac{\kappa f'(P_2^*) M_2^*}{(1+\kappa f(P_2^*))^2} \\ (1+\gamma g(P_1^*)) \left(\frac{1+\eta f(P_1^*)}{1+\kappa f(P_1^*)}\right) & 0 & h(P_1^*) M_1^* - \pi - D & D \\ 0 & (1+\gamma g(P_2^*)) \left(\frac{1+\eta f(P_2^*)}{1+\kappa f(P_2^*)}\right) & D & h(P_2^*) M_2^* - \pi - D \end{pmatrix}, \quad (7)$$

where $f(x) = \dfrac{x^n}{x^n + \theta_1^n}$, $g(x) = \dfrac{x^\nu}{x^\nu + \theta_2^\nu}$, $f'(x) = n\dfrac{\theta_1^n x^{n-1}}{(x^n + \theta_1^n)^2}$, $g'(x) = \nu\dfrac{\theta_2^\nu x^{\nu-1}}{(x^\nu + \theta_2^\nu)^2}$, and
$h(x) = \left(\frac{1+\eta f(P_1^*)}{1+\kappa f(P_1^*)}\right)\gamma g'(P_1^*) + (1 + \gamma g(P_1^*))\left(\frac{(\eta-\kappa)f'(P_1^*)}{(1+\kappa f(P_1^*))^2}\right)$.

In this way, it is possible to identify regions of parameter space that are enriched for symmetry breaking in the absence of transport bias.

References

1. Cummins B, Gedeon T, Harker S, Mischaikow K, Mok K. Combinatiorial representation of parameter space for switching networks. SIAM J. on Appl. Dyn. Syst. 2016; 15(4):2176–2212

2. Cummins B, Gedeon T, Harker S, Mischaikow K. DSGRN: Examining the dynamics of families of logical models. Front. Physiol. 2018; 9(549):1–8.

3. Baire RL. Sur les fonctions de variables réelles. Ann. di Mat. 1899; 3(1):1–123.

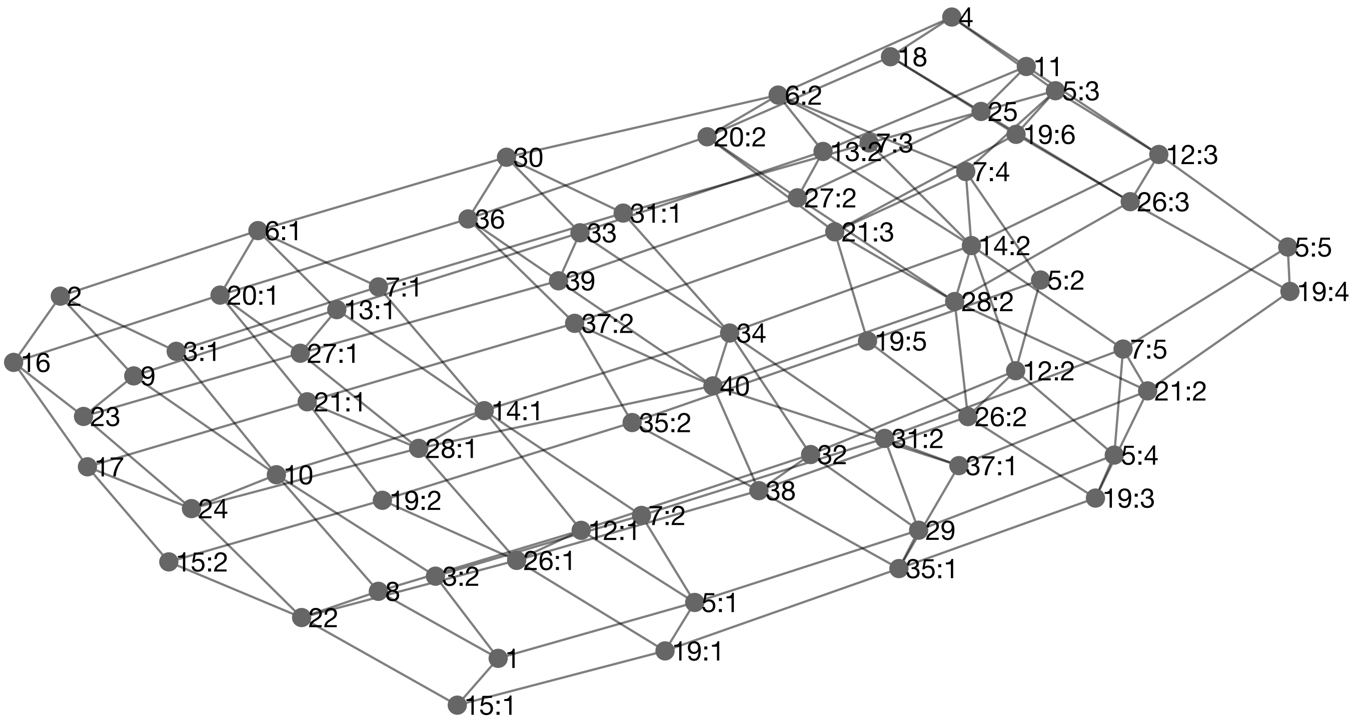

Fig A. Adjacency graphs of parameter space for $\theta_2 < \theta_1$. Connectivity of all regions identified through the division of the entire parameter space under the sharp switch limit.

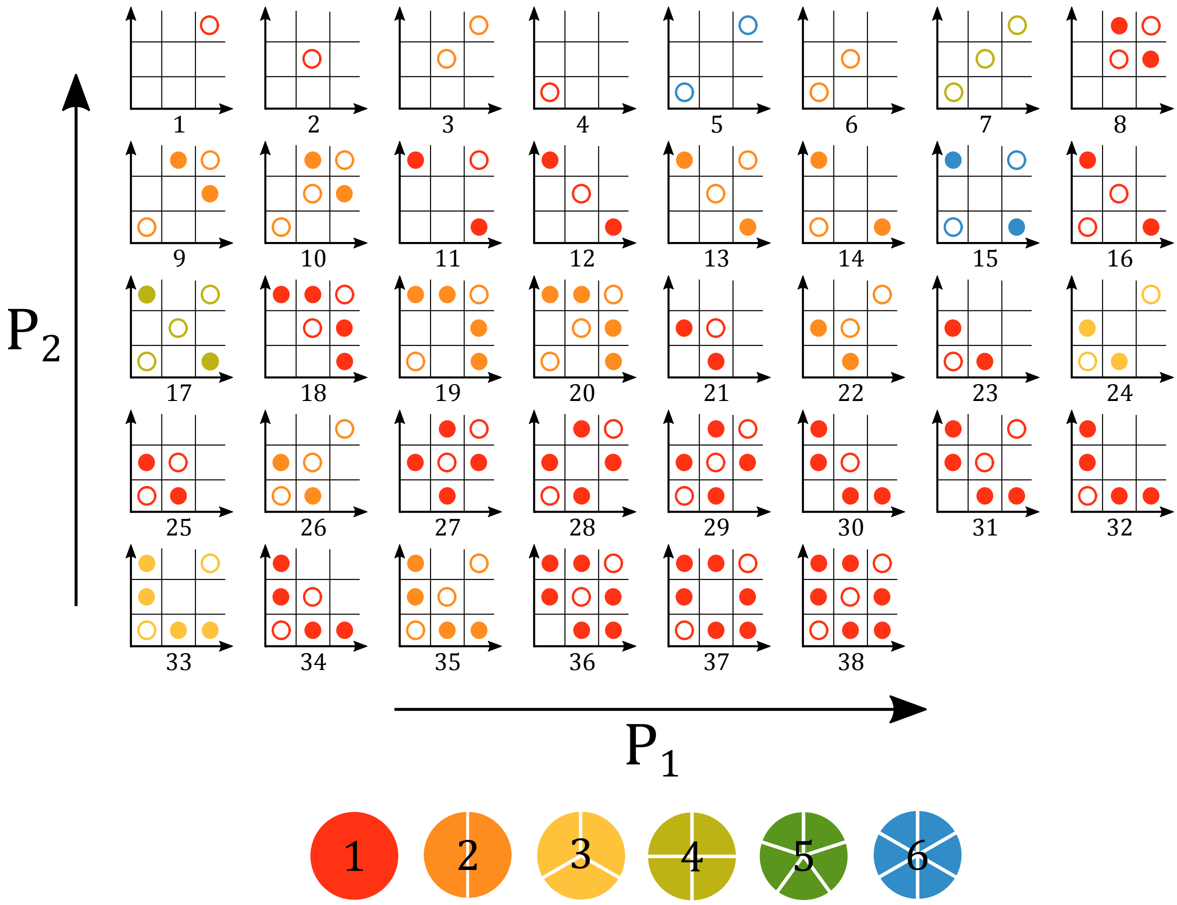

Fig B. Distribution of steady states in the sharp switch limit for $\theta_1 < \theta_2$. Each distinct parameter region is defined by its distribution of attractors within the 9 regions of P_1, P_2 space), with symmetric steady states denoted by hollow dots and asymmetric steady states denoted as filled dots. The color represents the number of disjoint decomposition sets that exist throughout parameter space that contain the same steady state distribution.

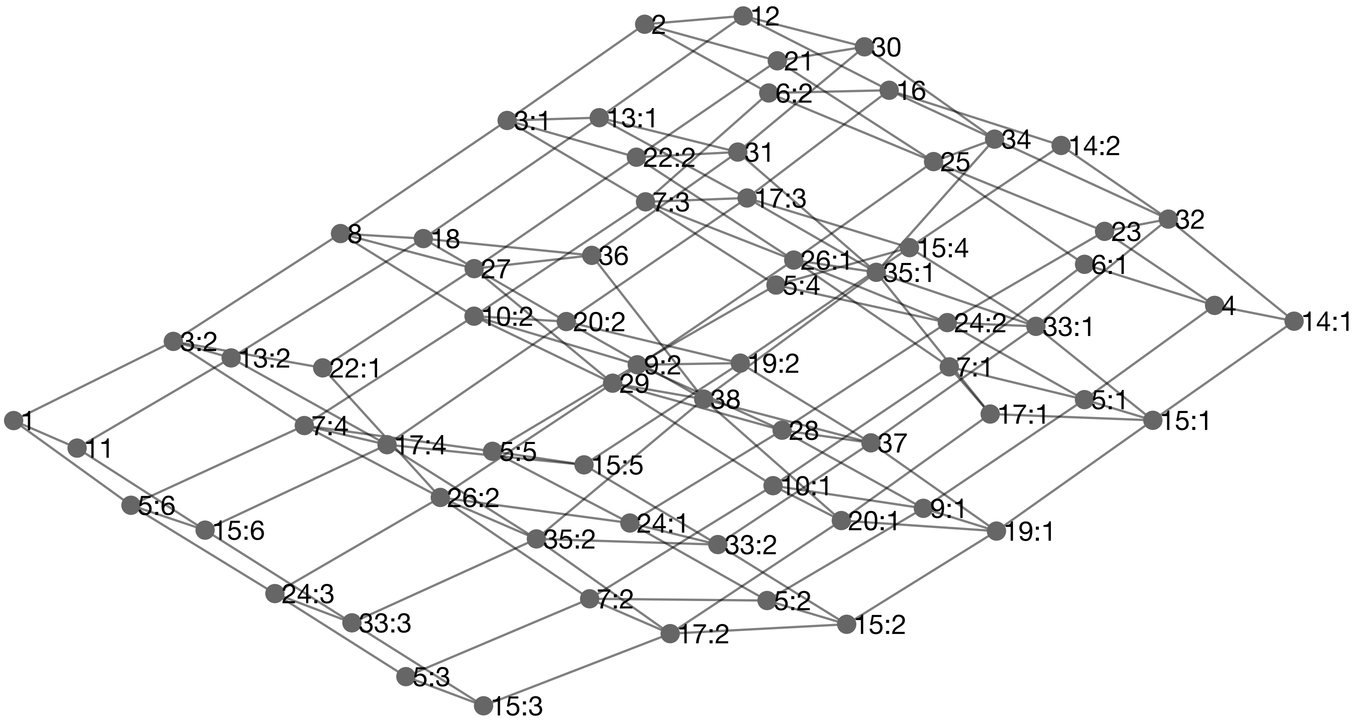

Fig C. Adjacency graph of parameter space for $\theta_1 < \theta_2$. Connectivity of all regions identified through the division of the entire parameter space under the sharp switch limit.

Table A. Parametric inequalities defining regions of P_1, P_2 space for the $\theta_2 < \theta_1$ case. The relationships between P_1, P_2 and θ_1, θ_2 define the values for each Heaviside expression for each steady state in each region. Here, by symmetry, the inequalities defined by conjugate spatial regions (1 & 3, 2 & 6, and 5 & 7) are equivalent.

**Region**	**Definition**	**Inequalities**
**0**	$P_1 < \theta_2 < \theta_1$<br>$P_2 < \theta_2 < \theta_1$	$\frac{1}{\pi\mu} < \theta_2$
**1**	$\theta_2 < P_1 < \theta_1$<br>$P_2 < \theta_2 < \theta_1$	$\theta_2 < \frac{(1+\gamma)(\pi+\epsilon)+\epsilon}{\pi\mu(\pi+2\epsilon)} < \theta_1$<br>$\frac{(1+\gamma)\epsilon+(\pi+\epsilon)}{\pi\mu(\pi+2\epsilon)} < \theta_2$
**2**	$\theta_2 < \theta_1 < P_1$<br>$P_2 < \theta_2 < \theta_1$	$\theta_1 < \frac{(1+\gamma)(1+\eta)(2+\mu)(\pi+\epsilon)+(2+\mu(1+\kappa))\,\epsilon}{\pi\mu(\pi+2\epsilon)\,((1+\mu)(1+\kappa)+1)}$<br>$\frac{(1+\gamma)(1+\eta)(2+\mu)\epsilon+(2+\mu(1+\kappa))\,(\pi+\epsilon)}{\pi\mu(\pi+2\epsilon)\,((1+\mu)(1+\kappa)+1)} < \theta_2$
**3**	$P_1 < \theta_2 < \theta_1$<br>$\theta_2 < P_2 < \theta_1$	$\frac{(1+\gamma)\epsilon+(\pi+\epsilon)}{\pi\mu(\pi+2\epsilon)} < \theta_2$<br>$\theta_2 < \frac{(1+\gamma)(\pi+\epsilon)+\epsilon}{\pi\mu(\pi+2\epsilon)} < \theta_1$
**4**	$\theta_2 < P_1 < \theta_1$<br>$\theta_2 < P_2 < \theta_1$	$\theta_2 < \frac{1+\gamma}{\pi\mu} < \theta_1$
**5**	$\theta_2 < \theta_1 < P_1$<br>$\theta_2 < P_2 < \theta_1$	$\theta_1 < \frac{(1+\gamma)(1+\eta)(2+\mu)(\pi+\epsilon)+(1+\gamma)\,(2+\mu(1+\kappa))\,\epsilon}{\pi\mu(\pi+2\epsilon)\,((1+\mu)(1+\kappa)+1)}$<br>$\theta_2 < \frac{(1+\gamma)(1+\eta)(2+\mu)\epsilon+(1+\gamma)\,(2+\mu(1+\kappa))\,(\pi+\epsilon)}{\pi\mu(\pi+2\epsilon)\,((1+\mu)(1+\kappa)+1)} < \theta_1$
**6**	$P_1 < \theta_2 < \theta_1$<br>$\theta_2 < \theta_1 < P_2$	$\frac{(1+\gamma)(1+\eta)(2+\mu)\epsilon+(2+\mu(1+\kappa))\,(\pi+\epsilon)}{\pi\mu(\pi+2\epsilon)\,((1+\mu)(1+\kappa)+1)} < \theta_2$<br>$\theta_1 < \frac{(1+\gamma)(1+\eta)(2+\mu)(\pi+\epsilon)+(2+\mu(1+\kappa))\,\epsilon}{\pi\mu(\pi+2\epsilon)\,((1+\mu)(1+\kappa)+1)}$
**7**	$\theta_2 < P_1 < \theta_1$<br>$\theta_2 < \theta_1 < P_2$	$\theta_2 < \frac{(1+\gamma)(1+\eta)(2+\mu)\epsilon+(1+\gamma)\,(2+\mu(1+\kappa))\,(\pi+\epsilon)}{\pi\mu(\pi+2\epsilon)\,((1+\mu)(1+\kappa)+1)} < \theta_1$<br>$\theta_1 < \frac{(1+\gamma)(1+\eta)(2+\mu)(\pi+\epsilon)+(1+\gamma)\,(2+\mu(1+\kappa))\,\epsilon}{\pi\mu(\pi+2\epsilon)\,((1+\mu)(1+\kappa)+1)}$
**8**	$\theta_2 < \theta_1 < P_1$<br>$\theta_2 < \theta_1 < P_2$	$\theta_1 < \frac{(1+\gamma)(1+\eta)}{\pi\mu(1+\kappa)}$

Table B. Parametric inequalities defining regions of P_1, P_2 space for the $\theta_1 < \theta_2$ case. The relationships between P_1, P_2 and θ_1, θ_2 define the values for each Heaviside expression for each steady state in each region. Here, by symmetry, the inequalities defined by conjugate spatial regions (1 & 3, 2 & 6, and 5 & 7) are equivalent.

Region	Definition	Inequalities
**0**	$P_1 < \theta_1 < \theta_2$<br>$P_2 < \theta_1 < \theta_2$	$\frac{1}{\pi\mu} < \theta_1$
**1**	$\theta_1 < P_1 < \theta_2$<br>$P_2 < \theta_1 < \theta_2$	$\theta_1 < \frac{(1+\eta)(2+\mu)(\pi+\epsilon)+(2+\mu)\epsilon}{\pi\mu(\pi+2\epsilon)\left((1+\mu)(1+\kappa)+1\right)} < \theta_2$<br>$\frac{(1+\eta)(2+\mu)\epsilon+(2+\mu)(\pi+\epsilon)}{\pi\mu(\pi+2\epsilon)\left((1+\mu)(1+\kappa)+1\right)} < \theta_1$
**2**	$\theta_1 < \theta_2 < P_1$<br>$P_2 < \theta_1 < \theta_2$	$\theta_2 < \frac{(1+\gamma)(1+\eta)(2+\mu)(\pi+\epsilon)+\left(2+\mu(1+\kappa)\right)\epsilon}{\pi\mu(\pi+2\epsilon)\left((1+\mu)(1+\kappa)+1\right)}$<br>$\frac{(1+\gamma)(1+\eta)(2+\mu)\epsilon+\left(2+\mu(1+\kappa)\right)(\pi+\epsilon)}{\pi\mu(\pi+2\epsilon)\left((1+\mu)(1+\kappa)+1\right)} < \theta_1$
**3**	$P_1 < \theta_1 < \theta_2$<br>$\theta_1 < P_2 < \theta_2$	$\frac{(1+\eta)(2+\mu)\epsilon+(2+\mu)(\pi+\epsilon)}{\pi\mu(\pi+2\epsilon)\left((1+\mu)(1+\kappa)+1\right)} < \theta_1$<br>$\theta_1 < \frac{(1+\eta)(2+\mu)(\pi+\epsilon)+(2+\mu)\epsilon}{\pi\mu(\pi+2\epsilon)\left((1+\mu)(1+\kappa)+1\right)} < \theta_2$
**4**	$\theta_1 < P_1 < \theta_2$<br>$\theta_1 < P_2 < \theta_2$	$\theta_1 < \frac{(1+\gamma)}{\pi\mu} < \theta_2$
**5**	$\theta_1 < \theta_2 < P_1$<br>$\theta_1 < P_2 < \theta_2$	$\theta_2 < \frac{(1+\gamma)(1+\eta)(\pi+\epsilon)+(1+\eta)\epsilon}{\pi\mu(\pi+2\epsilon)(1+\kappa)}$<br>$\theta_1 < \frac{(1+\gamma)(1+\eta)\epsilon+(1+\eta)(\pi+\epsilon)}{\pi\mu(\pi+2\epsilon)(1+\kappa)} < \theta_2$
**6**	$P_1 < \theta_1 < \theta_2$<br>$\theta_1 < \theta_2 < P_2$	$\frac{(1+\gamma)(1+\eta)(2+\mu)\epsilon+\left(2+\mu(1+\kappa)\right)(\pi+\epsilon)}{\pi\mu(\pi+2\epsilon)\left((1+\mu)(1+\kappa)+1\right)} < \theta_1$<br>$\theta_2 < \frac{(1+\gamma)(1+\eta)(2+\mu)(\pi+\epsilon)+\left(2+\mu(1+\kappa)\right)\epsilon}{\pi\mu(\pi+2\epsilon)\left((1+\mu)(1+\kappa)+1\right)}$
**7**	$\theta_1 < P_1 < \theta_2$<br>$\theta_1 < \theta_2 < P_2$	$\theta_1 < \frac{(1+\gamma)(1+\eta)\epsilon+(1+\eta)(\pi+\epsilon)}{\pi\mu(\pi+2\epsilon)(1+\kappa)} < \theta_2$<br>$\theta_2 < \frac{(1+\gamma)(1+\eta)(\pi+\epsilon)+(1+\eta)\epsilon}{\pi\mu(\pi+2\epsilon)(1+\kappa)}$
**8**	$\theta_1 < \theta_2 < P_1$<br>$\theta_1 < \theta_2 < P_2$	$\theta_2 < \frac{(1+\gamma)(1+\eta)}{\pi\mu(1+\kappa)}$
